# Diagnostic value of circRNAs as effective biomarkers in human cardiovascular disease: an updated meta-analysis

**DOI:** 10.7150/ijms.67094

**Published:** 2022-02-07

**Authors:** Zhexiao Zhang, Runmin Guo, Yuhui Wang, Hairong Huang, Jie Liu, Chenfei Wang, Hongfu Wu, Tangbin Zou

**Affiliations:** 1 Key Laboratory of Research in Maternal and Child Medicine and Birth Defects, Guangdong Medical University, Foshan, China.; 2 Dongguan Key Laboratory of Environmental Medicine, School of Public Health, Guangdong Medical University, Dongguan, China.; 3 Department of Surgery, the Third Affiliated Hospital of Guangdong Medical University (Longjiang Hospital of Shunde District), Foshan, China.; 4 Key Laboratory of Stem Cell and Regenerative Tissue Engineering, Guangdong Medical University, Dongguan, China.

**Keywords:** circRNAs, cardiovascular disease, diagnosis, biomarker, meta-analysis

## Abstract

**Background:** A growing body of literature has demonstrated that circular RNAs (circRNAs) are the potential biomarkers in human cardiovascular disease (CVD). Therefore, a meta-analysis based on current studies was accomplished to appraise the role of circRNAs in the diagnostic of CVD patients.

**Methods:** Studies before October 30, 2021, were searched using PubMed, EMBASE, the Web of Science, and Cochrane Library. The diagnostic odds ratio (DOR) with a confidence interval (CI) of 95% was used to investigate the associations between circRNAs and CVDs.

**Results:** A total of 27 eligible articles were selected, including 47 studies, with 6833 participants meeting the criteria standard constrain. The pooled overall sensitivity and specificity for circRNAs expression profile in differentiating CVD patients from controls (non-CVDs or healthy subjects) were 0.81 (95%CI 0.78-0.83) and 0.74 (95%CI 0.68-0.78), respectively; the overall positive likelihood ratio was 3.1 (95%CI 2.5-3.7); the negative likelihood ratio was 0.26 (95%CI 0.22-0.31); the overall diagnostic odds ratio corresponding to an area under the curve of 0.85 (95%CI 0.81-0.88) was 12 (95%CI 9-16). Subgroup analysis indicated that the serum rather than blood has higher diagnostic accuracy. Likewise, meta-regression analysis demonstrated that the specimen, detection method, sample size, and publication year were the main sources of heterogeneity. Sensitivity analysis and Deeks' funnel plot revealed that our results are relatively robust.

**Conclusions:** Our evidence-based analysis results suggested that circRNAs provide higher diagnostic accuracy in the prediction of CVDs. Thus, circRNAs might be potential biomarkers in CVDs.

## Introduction

The concerns of epidemiology in the 20^th^ century gradually shifted with the decrease in the mortality and disability rate and the increase in non-communicable diseases 1. Among non-communicable diseases, cardiovascular disease (CVD) is the most severe public health problem, threatening the health of the general population 2. CVDs are characterized by high morbidity, high disability rate, high mortality, high recurrence rate, and severe complications 3. Therefore, CVDs have become one of the main causes of mortality around the world. With CVDs being such a huge burden, more and more genetic biomarkers of CVDs have been discovered, among which circRNAs have received considerable attention owing to their biological and clinical application in the diagnosis and treatment of CVDs 4,5.

CircRNA is a particular type of noncoding RNA with a closed circular structure, which is abundant in blood or tissue and acts as an indispensable member in a variety of biological processes 6-8. CircRNA is widespread in human cells, with the expression abundance sometimes even exceeding 10 times of their linear transcripts 9. It is formed by precursor mRNA (pre-mRNA) back-splicing, which is different from the classical shearing mode of general linear RNA 10. Due to various cyclization forms, it can be divided into three categories: exonic circRNA, intronic circRNA, and exon-intron circRNA 11,12. With the furthering research, several essential functions have been identified at the transcriptional and posttranscriptional levels, including acting as microRNA (miRNA) sponges, manipulating RNA binding proteins (RBPs), and controlling alternative splicing and parental gene expression 13. Generally, current studies have confirmed that the role of miRNA sponge is the main mechanism of circular RNA in disease 14.

CircRNAs have covalently closed loop structures with neither 5'-3' polarities nor polyadenylated tails, which leads to its various characteristics: stability, specificity and conservatism. Based on these characteristics, circRNAs have advantages over other types of RNAs in the diagnosis of specific diseases. Simultaneously, increasing studies have demonstrated that circRNAs are related to the occurrence and development of various diseases. In the cardiovascular system, circRNAs are not only important for the development of the heart and blood vessels 15, but they also play a vital role in the pathophysiology of heart abnormalities such as coronary artery disease (CAD), stroke, heart failure (HF), essential hypertension (EH), idiopathic pulmonary arterial hypertension (IPAH), acute Stanford type A aortic dissection (AAAD) and so on 16-18. Furthermore, under normal circumstances, the level of circRNAs in plasma and other body fluids is stable, suggesting that circRNAs have the potential to serve as diagnostic biomarkers for many diseases 19. For instance, circ-STAB2 and circCHFR play a crucial role in up-regulating the related target genes by sponging miR-939 and miR-370, respectively, thus playing a crucial vital role in atherosclerosis and CVDs 20,21. To date, a considerable number of biomarkers related to CVDs have been reported in the research while only a small part of circRNAs have been repeatedly verified and applied. Although it is doubtful whether circRNAs can be accurate and useful biomarkers for the diagnosis of CVDs, they have been extensively explored as biomarkers of cancer. Therefore, summarizing the connection between circRNAs and CVDs and providing new ideas are imperative. Herein, we summarize the latest research progress on circRNAs, focusing on exploring the accuracy of circRNAs-based biomarkers in the diagnosis of CVDs' outcomes, so as to demonstrate its role as a non-invasive diagnostic biomarker in this setting.

## Materials and methods

### Study selection and data collection

To get the high-quality research, systematic network document search was aimed at multiple website database, including PubMed, Embase, and the Web of science until October 30, 2021 , and the detailed search strategy were as follow: (a) 'circular RNA' or 'circRNA' and (b) 'Cardiovascular Disease' or 'Coronary Artery Disease' or 'Myocardial Infarction' or 'Heart Attack' or 'Coronary Heart Disease' or 'Ischemic Heart Disease' or 'Ischaemic Heart Disease' or 'Stroke' or 'Transient Ischemic Attack' or 'Transient Ischaemic Attack' or 'Vascular Accident' or 'Apoplexy' or 'High Blood Pressure' or 'Hypertension' or 'Atherosclerotic sclerosis' and (c) diagnosis or diagnostic or sensitivity or specificity or 'receiver operating characteristic curve. The relevant systematic reviews and references cited in the searched articles were also manually reviewed for additional studies of circRNAs in patients with CVDs. And all included studies were in English.

### Selected and removed criteria

The selected criteria were as follows: (a) studies patients were diagnosed with cardiovascular events (including Cardiovascular Disease, Coronary Artery Disease, Myocardial Infarction, Heart Attack, Coronary Heart Disease, Ischemic Heart Disease, Stroke, Transient Ischemic Attack, Transient Ischaemic Attack, Vascular Accident, Apoplexy, High Blood Pressure, Hypertension and Atherosclerotic Sclerosis); (b) expression of circRNAs was measured in the blood (serum, plasma, peripheral whole blood and leukocyte in peripheral blood); (c) the study has clinical results related to circRNAs expression; (d) AUC, sensitivity, specificity or 95% CI between circRNAs expression could be picked up directly or calculated indirectly in the study; (e) case-control studies were included.

The exclusion criteria were: (a) repeated research publication; (b) content has nothing to do with CVDs or circRNAs; (c) non-human studies, letters, experiments, reviews, meta-analysis, etc.

### Data extraction and quality evaluation

Two investigators independently extracted the information and data from all selected studies through cross-check. Any difference between investigators was discussed and resolved by a third investigator. The information and data were collected by a purpose-designed form: author, year of publication, country, the type of CVDs, the number of patients and controls, specimens, the basic information of circRNAs, and more. We collected the numbers of true-positive (TP), false-positive (FP), true-negative (TN), false-negative (FN), platform, fold changes (FC), cut-off criteria of the dysregulated circRNAs through original data. ∣FC∣ ≥ 1.5, *P* < 0.05 were selected as the cut-off threshold. The quality of studies was evaluated using the Quality Assessment of Diagnostic Accuracy Studies (QUADAS-2) checklist.

However, there is still no unified naming standard for circRNAs, and different discoverers will use their own ways to name newly discovered circRNAs. Different circRNAs with different expressions will cause difficulties in the comparative analysis of circRNAs in CVDs. One needs to merge combine information to better unify circRNAs. Referring to the naming methods in the literature, we use the Gene symbol of the corresponding circRNAs to unify the naming format, which is convenient for finding potential biomarkers of CVDs. “Gene symbol” represents the name of the gene where the circRNAs is located. Therefore, we found the corresponding gene symbol by referring to the circRNAs database mentioned in the literature or circbase (http://www.circbase.org/). If the gene symbol still could not be found in the original literature data and circbase, the original circRNAs name will be retained (hsa_circ_0141720 and hsa-circRNA9102-5).

### Statistical analysis

Stata 12.0, RevMan 5.0, and Meta-Disc 1.4 softwares were used for all statistical analyses. The sensitivity, specificity, positive, negative likelihood ratio (LR^+^ and LR^-^), and diagnostic odds ratio (DOR) of each circRNAs about the diagnostic value of CVD events were worked out from each study. The summary receiver operating characteristic (SROC) was curved to measure the overall performance of circRNAs in differentiating CVD patients from the controls by calculating the area under the curve (AUC). The heterogeneity among the qualified studies was indicated by the Cochran Q-test and Inconsistency Index (*I^2^*), in which *I^2^* > 50 implied significant heterogeneity. If *I^2^* <50% or *P*>0.5, then the random-effects model (Der Simonian and Laird) was applied. Or else, we selected the fixed-effects model (Mantel-Haenszel). Subgroup analysis, sensitivity analysis, and meta-regression should be carried out to explore the potential causes of heterogeneity. Additionally, publication bias was assessed by Deeks' funnel plots asymmetry test, and *P* < 0.05 indicated remarkable bias.

## Results

### Literatures search results and characteristics of the selected studies

**Figure 1:**
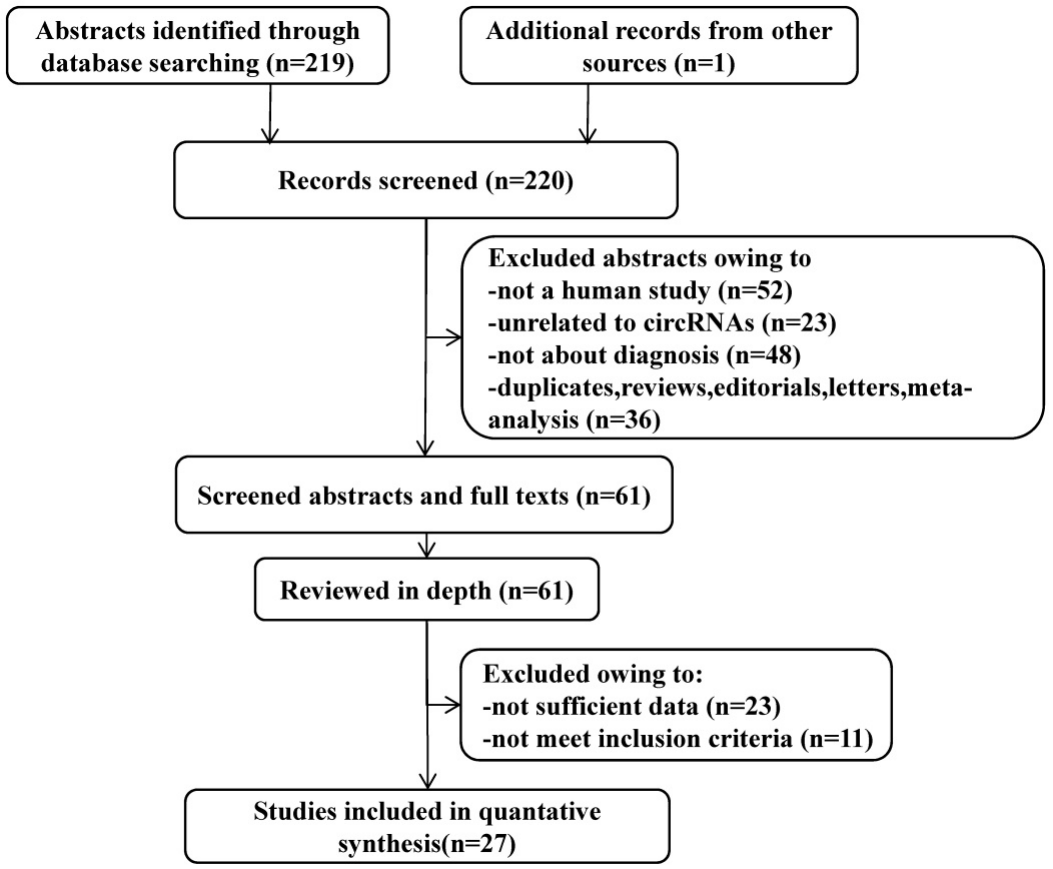
A flow diagram demonstrating the study selection process. The process of study selection including identification, screening, eligibility extraction, and inclusion steps were depicted in the flow diagram. Out of 220 records identified from three databases, 27 studies met the selection criteria.

After the initial search algorithm, a total of 219 articles were retrieved from PubMed, MEDLINE, and the Cochrane database (Figure 1), and 1 article was included by manually searching the publication bibliography and review articles. Among them, 36 articles were duplicates, reviews, letters, or meta-analysis, 52 articles were not human studies, 23 articles were unrelated to circRNAs and 48 articles were not about the diagnosis. After these articles were removed, 61 articles were further evaluated. The full-text review identified 23 articles without sufficient data and 11 articles that did not meet the inclusion criteria. Finally, 27 articles, including 47 eligible studies, were included in this meta-analysis (Table 1).

**Figure 2:**
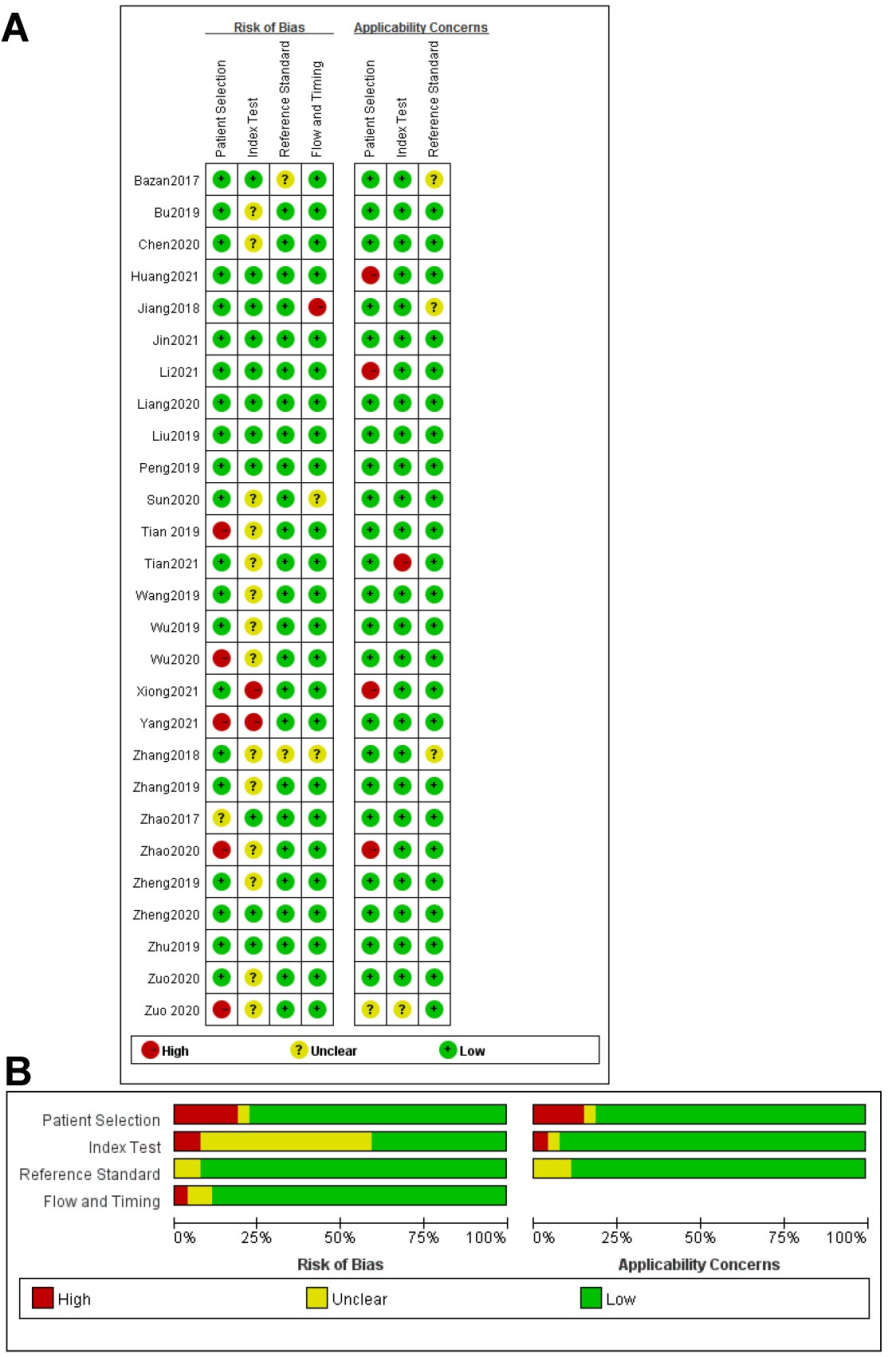
Overall quality assessment of eligible studies by QUADAS-2 tool. **A.** Methodological quality summary (by study). **B.** Methodological quality graph (overall).

All of these studies were published from January 1, 2010, to October 30, 2021, containing 6833 participants (Table 2). Among them, only one was from the USA, the rest were from China. In this meta-analysis, 10 different types of CVDs were contained, namely, 8 studied strokes, 15 CADs, 3 EHs, 3 HF, 4 MI, 2 PE, 1 AAAD, 2 KD, 2 PoAF and 8 PAH. PCR (qPCR), RT-PCR, or qRT-PCR was used for quantitative detection of these circRNAs. A total of 39 circRNAs were investigated in these articles, of which 29 were up-regulated and 10 circRNAs were down-regulated. Participants' blood was the main specimen source. According to the result of the QUADAS-2 assessment, the included 27 articles were at a certain risk of bias regarding patient selection, index test, and flow and timing. Figure 2A shows the risk degree of bias risk and applicability of each study in detail, while Figure 2B uses a bar chart to visually show the ratio of bias risk and applicability risk of the overall included literatures (Figure 2A, 2B). As we can see from the figures, the main causes of these risks of bias and application concerns were the blinding of participants, a lack of information on exclusion criteria and not meeting the prior undetermined threshold.

### Diagnostic performance

**Figure 3:**
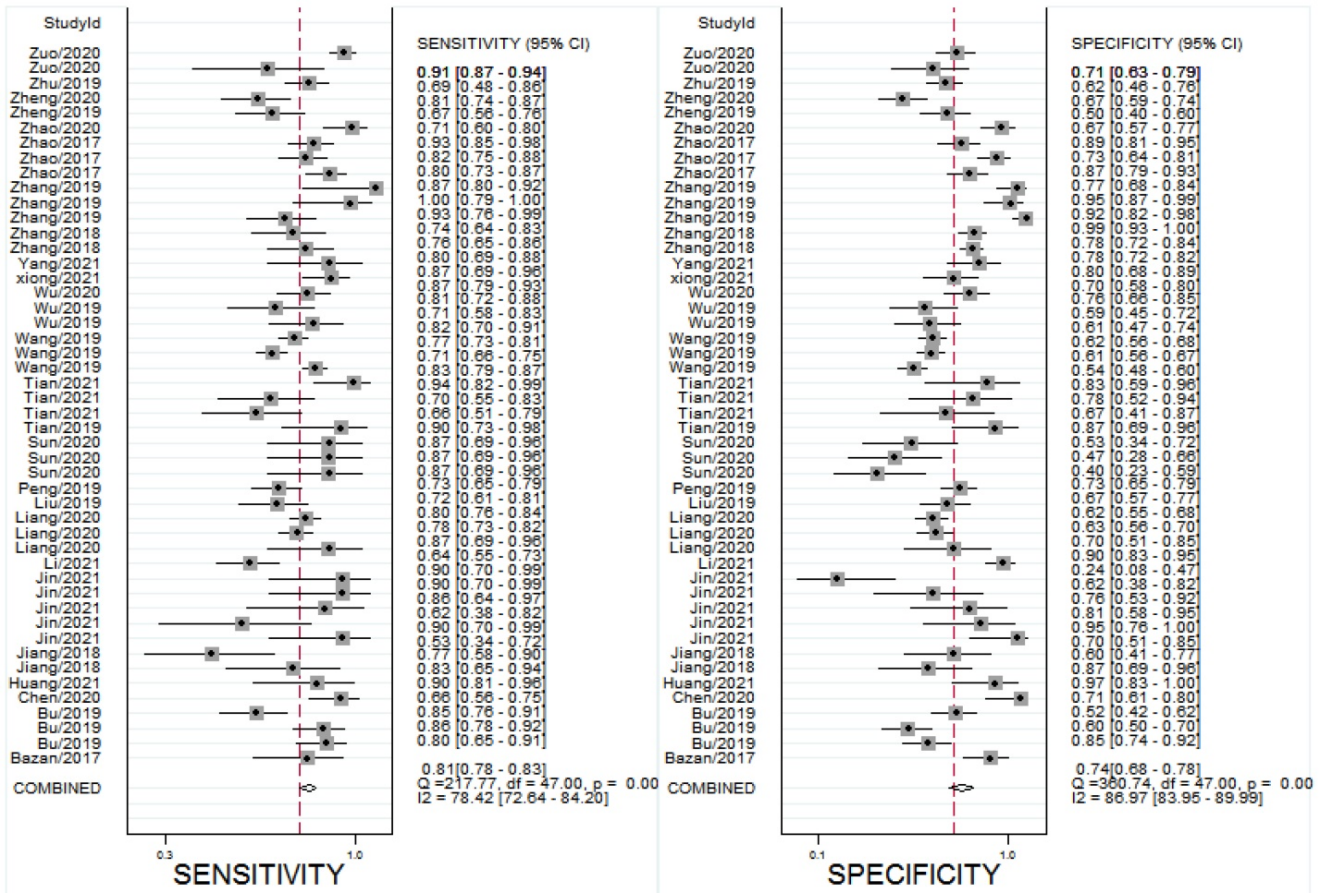
Forest plots for studies on overall circRNAs used in the diagnosis of CVDs among 27 studies included in the meta-analysis. **(A)** Sensitivity of circRNAs in diagnosis of CVDs and **(B)** specificity of circRNAs in diagnosis of CVDs.

**Figure 4:**
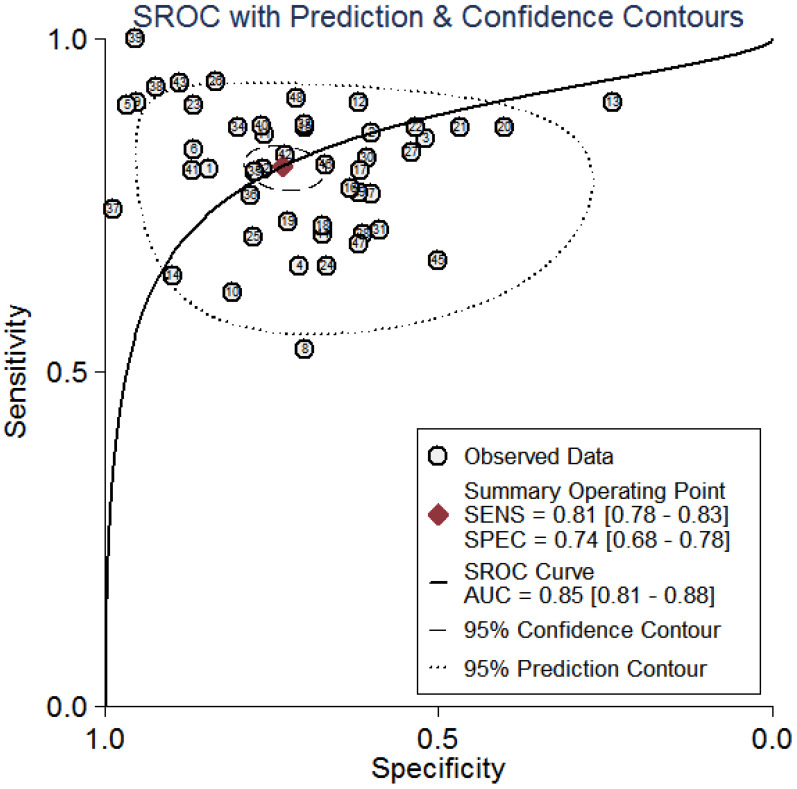
Summary receiver operator characteristic curves (SROC) of circRNAs for the diagnosis of CVDs in overall population.

All the results of individual studies were summarized to understand the potential application of circRNAs in the diagnosis of CVDs. The Spearman correlation coefficient was 0.282 (*P*=0.052), indicating no threshold effect in the accuracy estimates of circRNAs. Forest plots of the sensitivity and specificity of circRNAs for diagnosing CVDs are displayed (Figure 3). The random-effects model was employed to estimate the combined effects in virtue of the significant heterogeneity (*I*²=78.42% and 86.97%). The indexes are as follows: sensitivity, 0.81 (95%CI 0.78-0.83); specificity, 0.74 (95%CI 0.68-0.78); the pooled LR^+^, LR^-^ and DOR were 3.1 (95%CI 2.5-3.7), 0.26 (95%CI 0.22-0.31), and 12 (95%CI 9-16), respectively. Comprehensive diagnostic performance was evaluated by the summary receiver operating characteristic curve (SROC). The area under the SROC can accurately reflect the authenticity of the diagnostic test. The closer the area under the SROC is to 1, the better the authenticity of the diagnostic test is. The AUC was 0.85 (95%CI 0.81-0.88), which means that the overall circRNAs has good diagnostic accuracy (Figure 4). Furthermore, a series of analyses, including subgroup analysis, sensitivity analysis, meta-regression, and publication bias, were conducted to explore the potential sources of heterogeneity between studies.

### Subgroup analysis

Subgroup analyses based on the specimen, disease types, detection method, sample size, source of control, and published year were conducted. The main results of subgroup analyses were summarized (Table 3). Furthermore, we drew an SROC curve to evaluate the accuracy of the diagnosis (Supplementary Figure S1). From the perspective of disease types, the diagnostic value of CADs (DOR 8 (95%CI 6-11)) was lower than IS (DOR 20 (95%CI 8-50)). Compared with the blood sample, the specimen of serum has a higher overall diagnostic accuracy, indicating that circRNAs were serum-specific with extremely high diagnostic value. The subgroups analysis results of the detection method demonstrated that the diagnostic accuracy of qRT-PCR was not different from that of the qPCR test. In the stratified analysis based on the source of control, healthy subjects and non-CVDs subjects have similar recognition rates of identifying CVD patients with similar sensitivity, specificity, PLR, NLR, DOR and AUC. Studies using small sample size (<200) presented better diagnostic accuracy than those using large sample size (≥200), with increased DOR (14 vs. 9) and AUC (0.86 vs. 0.82). Similar results were observed in publication year (~2019: AUC = 0.84; 2019~: AUC = 0.84). Most groups still remained high heterogeneity, and only had a significant decrease in heterogeneity observed in qPCR and the year before 2019. In conclusion, significant differences occurred between CVD disease type, specimen, and sample size, while only slight differences were observed among the other subgroups.

### Sensitivity analysis, meta-regression analysis, and publication bias

**Figure 5:**
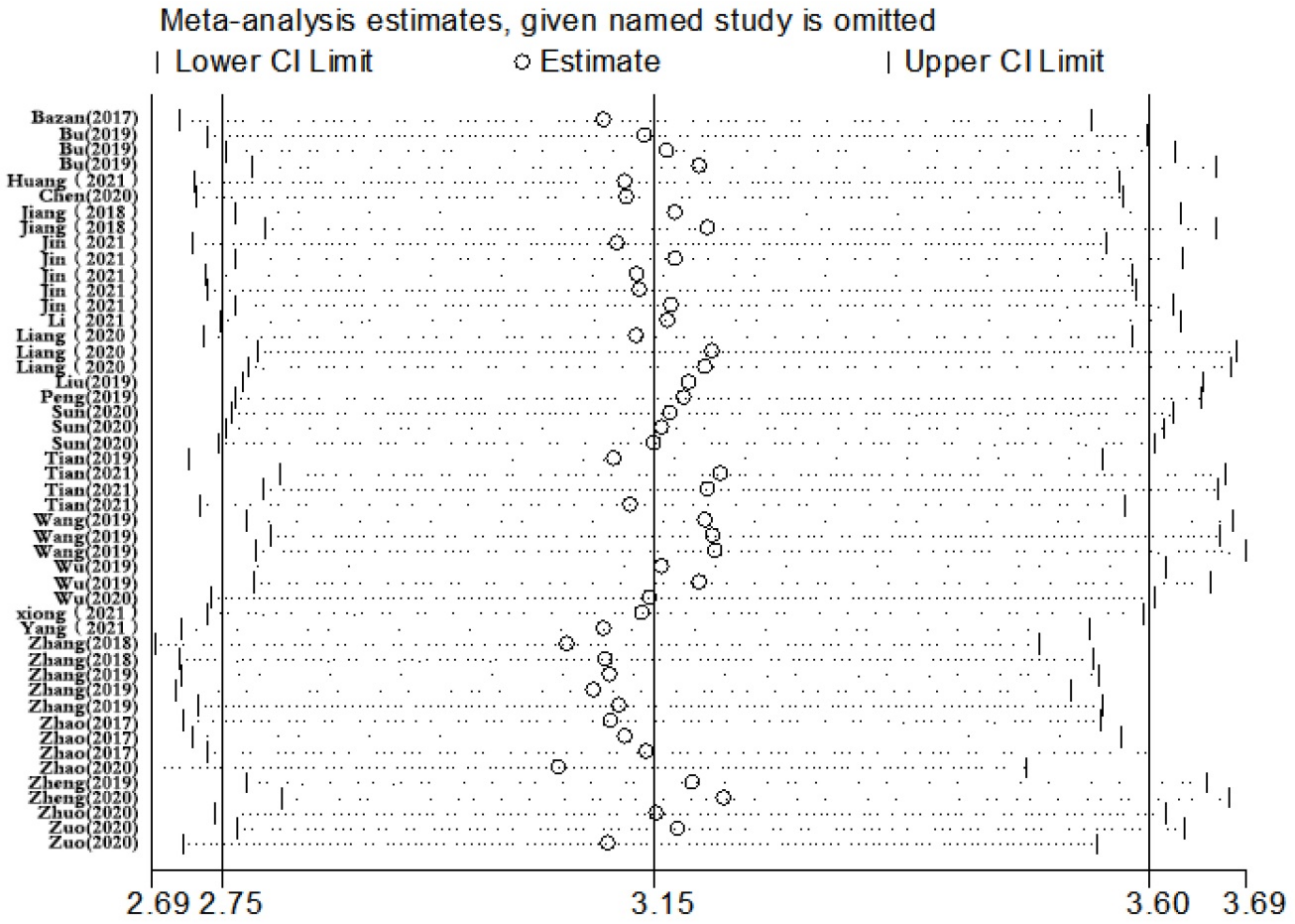
Sensitivity analysis of the result of the meta-analysis for CVDs.

**Figure 6:**
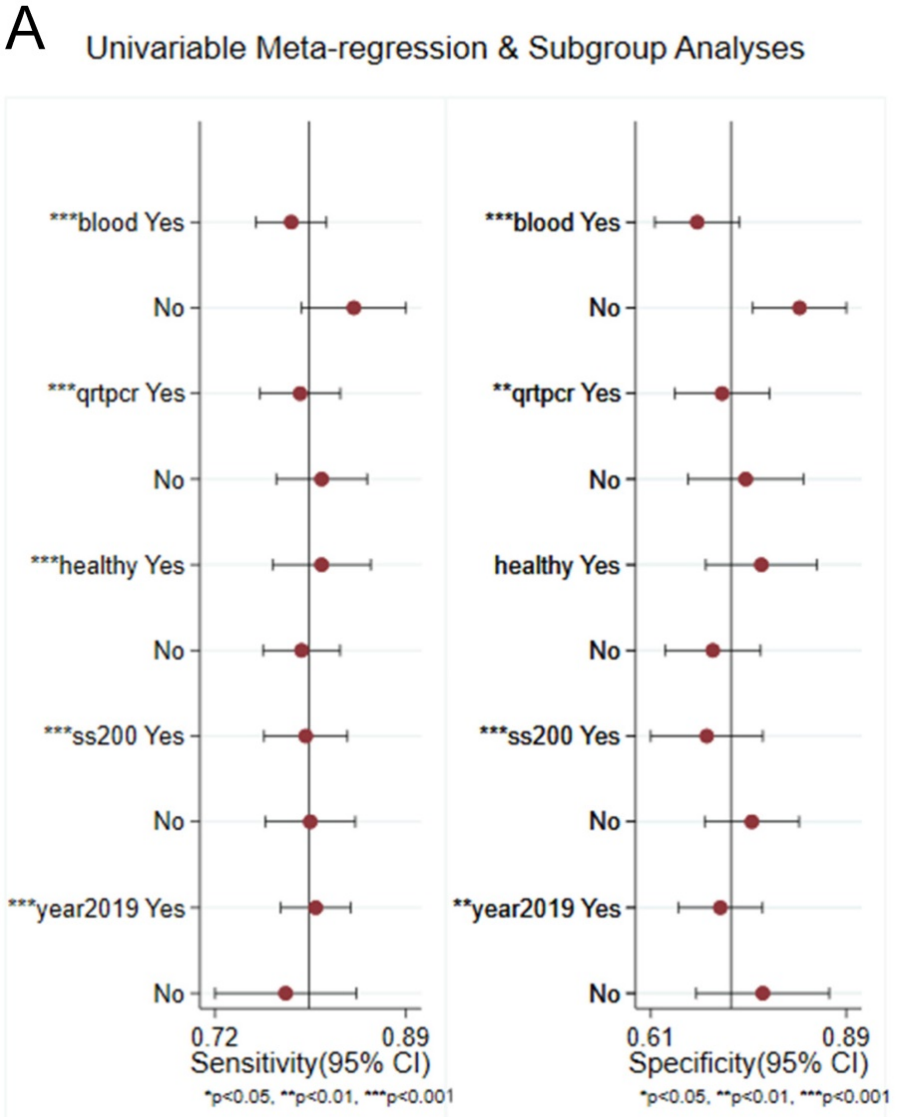
Univariable meta-regression for sensitivity and specificity of circRNAs for diagnosis of CVDs.

**Figure 7:**
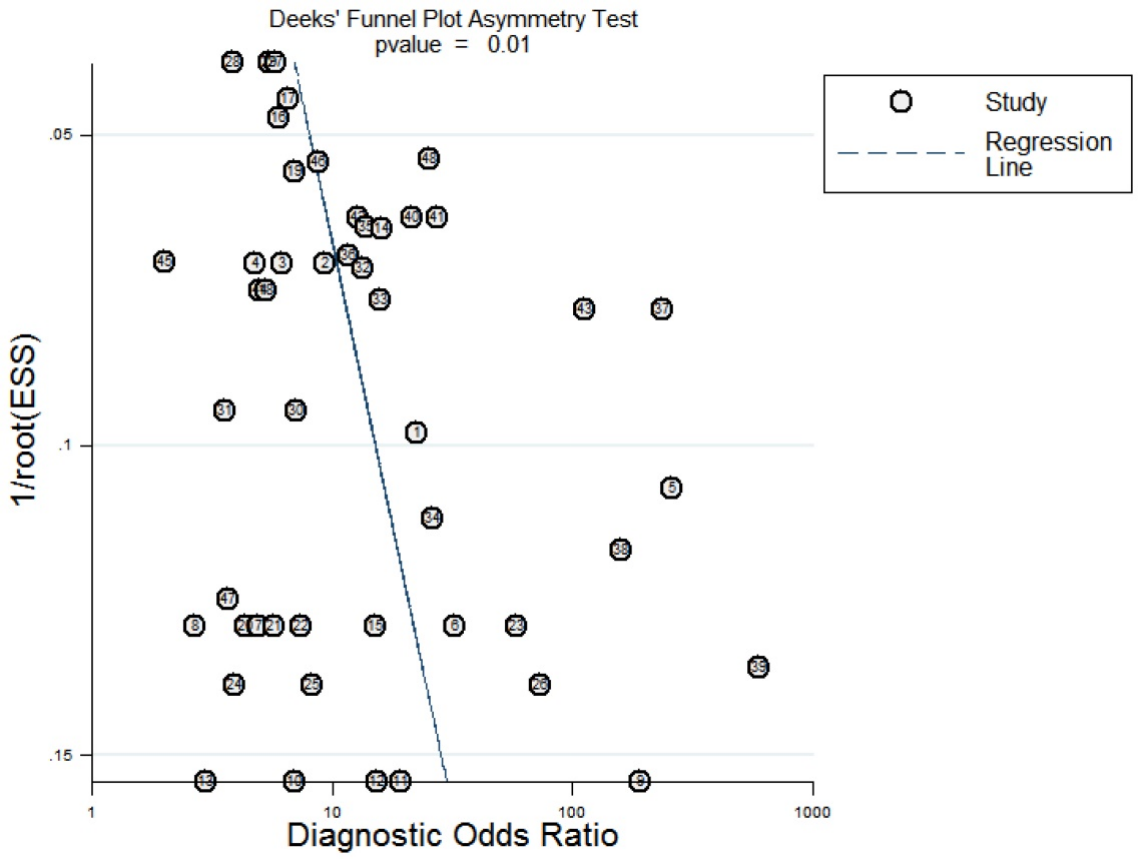
Deeks' funnel plot evaluating the potential publication bias of the included studies.

Sensitivity analysis was conducted simultaneously. One study was deleted at a time, and other studies were analyzed to estimate whether a single study would significantly affect the results. The results demonstrated that the pooled DOR in the result of the meta-analysis of CVDs were not significantly affected, indicating that this analysis confirmed the stability of our results (Figure 5). Subsequently, meta-regression analysis was performed on the bias of specimen, detection method, the source of control, sample size, and publication year (Figure 6). As illustrated in the figure, sensitivity was affected by all of these factors, whereas specificity was affected by specimen, detection method, sample size and publication year. Deeks' funnel plot asymmetry test was employed to assess publication bias, and showed a statistically significant value (*P*=0.01), reflecting data asymmetry and the possibility of publication bias (Figure 7).

### Clinical utility of index test

**Figure 8:**
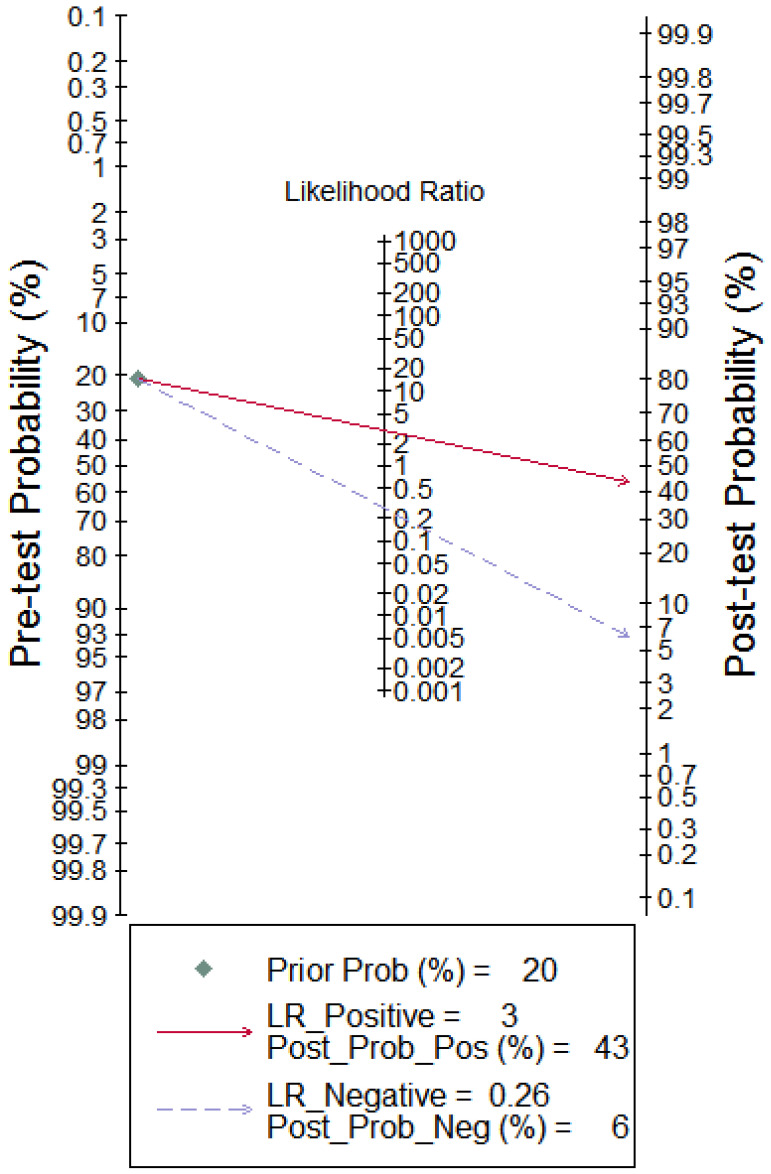
Fagan's nomogram evaluating the overall value of circRNAs for diagnosis of CVDs. The pre-test probability was set at 20%.

To better illustrate the clinical value of circRNA diagnosis in CVDs, Fagan's nomogram was adopted to calculate post-test probabilities. With a pre-test probability setting at 20%, the post-test probability increased to 43% with a positive LR of 3, while the post-test probability decreased to 6% with a negative LR of 0.26 (Figure 8). These results indicate that circRNAs can be good diagnostic biomarkers for CVDs.

## Discussion

With high morbidity and mortality rate, CVDs are the predominant cause of death around the world 49. Therefore, improving the efficiency of early diagnosis is crucial for the improvement in CVDs prognosis. CircRNAs have outstanding characteristics such as high selective abundance 50, high stability 51, conservation 52,53 and specific expression 19,54 in human body fluids and tumor tissues, making it a suitable biomarker for disease diagnosis. There is an abundance of literature on the roles of circRNA in the clinical diagnosis of various CVDs. The association between circRNAs and clinical outcomes needs to be further explored.

Our meta-analysis, comprising 27 articles that described 47 studies, with a total of 6833 participants (3449 cases and 3384 controls), can be considered the most comprehensive meta-analysis to evaluate the diagnostic value of circRNAs in CVDs. Within high diagnostic value, circRNAs represent a promising diagnostic biomarker for CVDs. According to our results, the pooled DOR of circRNAs was 12, demonstrating a powerful discriminating capacity of circRNAs for CVDs diagnosis. Among the circRNAs we analyzed, circST6GAL1 might serve as a biomarker of CVDs for the clinical outcome, because its serum expression in patients with CVDs was not only significantly higher than that in healthy volunteers, but also significantly correlated with the death 41. Simultaneously, the combined circRNAs will provide a better alternative, as more than two circRNAs would have better diagnostic value with significant sensitivity and specificity. The quality of our study was evaluated by the QUADAS-2 tool 55, with four domains of patients' selection, index tests, risk of bias, and flow and timing. The results of the quality assessment vary from low to high and may affect the stability of the combined results. The included studies were at high risk due to a lack of information on patient selection, exclusion criteria, or pre-specified thresholds, etc. Meanwhile, most of the literature used retrospective analysis, affecting the stability of the meta-analysis results. Therefore, it is urgent to make clear the specific situation of patient selection and adopt prospective research, so as to improve the results of these findings.

The pooled results revealed that there was a large degree of heterogeneity among the overall study. Then, the various causes of heterogeneity were clarified in our study. Spearman correlation coefficient was 0.282 (*P* = 0.052), suggesting that threshold effect was not the cause of heterogeneity. The results of the subgroup analyses study may provide some useful information for the high heterogeneity. As illustrated from the overall results, most of the subgroup analyses exhibited significant sensitivity and specificity heterogeneity (Table 3), except among studies detected by qPCR in specificity and published before 2019. Second, the subgroup analyses performed based on CVD revealed that the diagnostic value of circRNAs in patients with IS was higher compared with CAD. Due to the small sample size for other types of CVDs, we can't reach conclusions statistically. Both healthy subjects and non-CVDs controls can achieve the same diagnostic accuracy, demonstrating that circRNAs could be used for both population screening and as biomarkers to distinguish patients from non-CVDs controls. It can be seen from the results of subgroup analysis of the specimen, the serum is a better specimen in detecting circRNAs for CVDs. Therefore, the serum should be more widely used in clinical detection, such as blood biochemical tests and protein marker assays. Meanwhile, we made an interesting observation that combining multiple circRNAs or combining circRNAs with miRNAs yielded better diagnostic performance compared to single circRNAs in studied by Wang et al. 35 and Zheng et al. 45.

Additionally, a subsequent meta-regression analysis was performed. The results suggested that detection methods, the source of controls, sample size, and the publication year were not the origin of heterogeneity. Among them, the specimen is most likely to be the biggest factor affecting the heterogeneity. As with all meta-analysis, our study had potential publication bias with a statistically significant value, implying that better quality researches need to be included. Sensitivity analysis was also applied to analyze heterogeneity. However, no outlier studies were found, reflecting that our results were fairly reliable. Confounding factors such as age and gender have been statistically analyzed in the literature, and the risk factors have been excluded.

After searching, we found that a meta-analysis has been published on the diagnostic value of circRNAs in CVDs 56. However, our study exhibited superiority in the following two areas compared to the previous work. First, our sample size was larger than that and the research data has been updated. The data we collected is due in October 2021, while the last study was due in September 2018, indicating that our study is more robust and reliable. Moreover, our study observed a higher diagnostic odds ratio (DOR:12, (95%CI: 9-16) than the previous study (DOR:2.94, (95%CI: 2.35-3.85)). Next, various subgroup analyses such as disease types, detection method, sample size, source of control, and published year were conducted to provide more information on and to give a more comprehensive insight into the diagnostic role of circRNAs in CVDs.

At the end of the analysis, the potential target and related pathways of circRNAs in the database were collected (Table 4), revealing its internal mechanism of promoting the occurrence and development of CVDs. Part of the circRNAs we mentioned are involved in the metabolic pathway and the PI3K-Akt signaling pathway, which may be involved in the activation of metabolic regulation, hypertrophy, and survival pathways, essential for the cardiovascular system cells 57. By over-expressing hsa_circ_0004104 in THP-1-derived macrophages, it was revealed that atherosclerotic susceptibility genes, such as IDO1, MMP8, and CD40, were up-regulated, and anti-atherosclerotic genes, such as ApoA I, RNASE1, were down-regulated, indicating that hsa_circ_0004104 plays a crucial role in the pathogenesis of atherosclerosis 35. Some of the circRNAs, such as circ_0072309, can induce apoptosis and inhibit cell proliferation and migration by acting on endothelial cells or smooth muscle cells, leading to the formation of atherosclerosis. Other circRNAs can induce inflammation and cause abnormalities in endothelial function by promoting the abnormal expression of pro-inflammatory factors, chemokines, and adhesion molecules. Although related pathways between circRNAs and CVDs have been listed, the detailed relationship between them remains to be explored.

In real life, many patients suffering from CVDs are not diagnosed until the middle or late stage of the CVDs, delaying the best time for earlier treatment. Therefore, early diagnosis of CVDs is of great clinical significance. As non-invasive and pain-free biomarkers for detection, circRNAs may regulate cellular functions by competing with “sponge” miRNAs. Rather than the classical detection method, only serum or plasma, which has broad application prospects due to the characteristics of non-invasive, high acceptability, and high stability, is required for detection. Statistical analysis of the data demonstrated that combining circRNAs with existing clinical diagnostic methods, such as coronary angiography (CAG), routine electrocardiogram (ECG), and Holter monitoring, will achieve a higher diagnostic sensitivity and specificity compared to a single indicator. However, the research on the clinical application of circRNAs as biomarkers is still in fancy. Thus, turning this idea into reality is full of difficulties and challenges. First, the use of circRNAs as biomarkers has not been validated by enough experiments. Second, the specificity of circRNAs as biomarkers for CVDs still needs to be improved. For example, among the circRNAs collected in this meta-analysis, circZNF609 is used as a new biomarker for the diagnosis of CAD 29, Nevertheless, circZNF609 can also serve as a diagnostic biomarker for Hirschsprung disease (HSCR) 58. The expression levels of circRNAs are also downregulated in these diseases, generating a challenge for the diagnosis of a certain disease. Third, risk factors affecting the expression level of circRNAs, such as gender, age, environment,and diabetes, need to be further explored. Lastly, although a considerable number of circRNAs have been reported as biomarkers for the diagnosis of CVDs, only a few of them could be replicated. Thus, there is still a long way to go before circRNAs can be used as standard biomarkers of CVDs under a series of technical barriers and financial difficulties.

Research on the theoretical mechanism should be considered to realize the clinical application of circRNAs. Further studies are needed to confirm the safety and efficiency of circRNAs in diagnosis. Clinical trials of circRNAs as biomarkers should be conducted as soon as possible. Since further studies and rigorous experiments should be implemented, putting circRNA as biomarkers in clinical diagnosis still has a long way to go.

Although we strive to achieve a comprehensive and accurate analysis, several limitations remained, the main one being the significant heterogeneity among the included studies. Excessive heterogeneity cannot be completely eliminated, though subgroup analysis and meta-regression analysis have been conducted to explore the sources of heterogeneity. Notably, that specimen diversity and baseline differences between samples will affect the stability of our research results. Meanwhile, all kinds of CVDs are not the same, and unified analysis will lead to greater heterogeneity. The diagnostic value of circRNAs and specific types of CVDs should be further clarified. Secondly, only one study was conducted in American, and the rest studies were conducted in China, which will cause other ethnicities to be left out, leading to population selection bias. In addition, we have included studies with the small sample size, which may lead to publication bias. Small sample studies tend to report a higher effect value, though the research quality of small samples is often inferior to that of the large sample 59. Finally, the credibility of causality between circRNAs and CVDs needs to be increased by more cell culture or animal model studies. Moreover, these candidate circRNAs biomarkers from cells or animal models must be verified in patients to illustrate their clinical value. Therefore, more high-quality, well-designed studies are required to confirm our findings.

## Conclusion

Despite the limitations of the existing medical literature, research has verified that circRNAs can be useful biomarkers with advantages both in early screening and in distinguishing patients from non-CVD controls for its good diagnostic efficiency (AUC = 0.85). However, the feasibility, reliability, and repeatability of circRNA as next-generation CVD biomarkers should be fully verified by more studies.

## Supplementary Material

Supplementary figure.

## Figures and Tables

**Table 1:** Characteristics of 21 articles included in the meta-analysis

Study	Year	CVD	Patients (controls)	Source of control	Specimen	Method	Design type	CircRNA
Bazan 22	2017	IS	41(71)	Non-urgent	Serum	RT-PCR	Retrospective	circR-284(U)
Bu 23	2019	CAD	585(585)	Healthy	Blood	qRT-PCR	Retrospective	hsa_circ_0008507(U)hsa_circ_0001946(U) hsa_circ_0000284(D)
Chen 24	2020	AIS	80(30)	Healthy	Serum	qRT-PCR	Retrospective	hsa_circ_0141720(U)
Huang 25	2021	CAD	30(30)	Non-CAD	Blood	qRT-PCR	Retrospective	hsa_circ_0001946(U)
Jiang 26	2018	PE	35(35)	Non-PE	Blood	qRT-PCR	Prospective	hsa_circ_0004904(U)hsa_circ_0001855(U)
Jin 27	2021	PAH	21(21)	Healthy	Blood	qRT-PCR	Retrospective	hsa_circNFXL1_009(D) hsa_circMFN2_004(D) hsa_circ_ZNF302(D) hsa_circGSDMD_004(U) hsa_circWDR37_016(U)
Li 28	2021	IS	118(118)	Healthy	Blood	qRT-PCR	Retrospective	hsa_circ_0001599(U)
Liang 29	2020	CAD	330(209)	Non-CAD	Blood	qPCR	Retrospective	circZNF609(D)
Liu 30	2019	EH	89(89)	Healthy	Blood	qRT-PCR	Retrospective	hsa_circ_0126991(U)
Peng 31	2019	IS	160(160)	Non-IS	Blood	qPCR	Retrospective	circHECTD1(U)
Sun 32	2020	HF	30(30)	Non-HF	Plasma	qRT-PCR	Retrospective	hsa_circ_0062960(U)
Tian 33	2019	AAAD	30(30)	Non-AAAD	Serum	qRT-PCR	Retrospective	circMARK3(U)
Tian 34	2021	MI	47(18)	Healthy	Tissue	qRT-PCR	Retrospective	circSLC8A1(U) circNFIX(D)
Wang 35	2019	CAD	436(297)	Non-CAD	Blood	qRT-PCR	Retrospective	hsa_circ_0001879(U)hsa_circ_0004104(U)
Wu 36	2019	KD	56(56)	Healthy	Serum	qRT-PCR	Retrospective	circANRIL(D)hsa_circ_0123996(U)
Wu 37	2020	CAD	108(89)	Non-CAD	Plasma	qPCR	Retrospective	hsa_circ_0005540(U)
xiong 38	2021	CAD	109(70)	Non-CAD	Serum	qRT-PCR	Retrospective	circNPHP4(U)
Yang 39	2021	MI	30(60)	Non-MI	Blood	qRT-PCR	Retrospective	circRNA_104761(D)
Zhang 40	2018	PoAF	158(521)	Non-PoAF	Blood	qRT-PCR	Retrospective	hsa_circ_025016(U)
Zhang 41	2019	IPAH	82(82)	Healthy	Serum	qRT-PCR	Prospective	circ_0068481(U)
Zhao 42	2017	CAD	179(157)	Non-CAD	Blood	qRT-PCR	Retrospective	hsa_circ_0124644(U)
Zhao 43	2020	IS	75(90)	Non-IS	Blood	qRT-PCR	Retrospective	hsa_circ_0072309(D)
Zheng 44	2019	EH	89(89)	Healthy	Blood	qRT-PCR	Retrospective	hsa_circ_0014243(U)
Zheng 45	2020	EH	96(96)	Healthy	Blood	qRT-PCR	Retrospective	hsa-circRNA9102-5(U)
Zhu 46	2019	IS	170(170)	Non-IS	Blood	qPCR	Retrospective	circ-DLGAP4(D)
Zuo 47	2020	AIS	239(139)	Healthy	Blood	qRT-PCR	Retrospective	circFUNDC1+circPDS5B +circCDC14A(U)
Zuo 48	2020	AIS	26(42)	Non-AIS	Blood	qPCR	Retrospective	circFUNDC1(U)

Abbreviations: CAD, coronary artery disease; AIS, acute ischemic stroke; IS, ischemic stroke; PE, preeclampsia; HF, heart failure; EH, essential hypertension; IPAH, idiopathic pulmonary arterial hypertension; AAAD, acute stanford type A aortic dissection; KD, kawasaki disease; PoAF, postoperative atrial fibrillation; CVD, cardiovascular disease; circRNA, circular RNA; qPCR, real-time polymerase chain reaction; qRT-PCR, RT-PCR/qPCR combined technique; RT-PCR, reverse transcription-polymerase chain reaction; D, downregulated; U, upregulated.

**Table 2:** Characteristics of eligible studies included in the meta-analysis

Author	Year	CircRNAs	CVD	Platform	Cut-off criteria	TP	FP	FN	TN	SEN%	SPE%	AUC
Bazan	2017	circHIPK3 (1)	IS	RT-PCR	FC > 2, *P* < 0.05	33	11	8	60	80	85	0.91
Bu¹	2019	circGBA3 (2)	CAD	Microarray	FC > 2, *P* < 0.05	86	40	14	60	86	60	0.75
Bu²	2019	circCDR1 (3)	CAD	Microarray	FC > 2, *P* < 0.05	85	48	15	52	85	52	0.71
Bu³	2019	circHIPK3 (4)	CAD	Microarray	FC > 2, *P* < 0.05	66	29	34	71	66	71	0.68
Huang (2021)	2021	hsa_circ_0001946 (5)	CAD	qRT-PCR	FC > 2, *P* < 0.05	25	4	5	26	83.3	86.7	0.897
Chen	2020	hsa_circ_0141720 (6)	AIS	Microarray	FC > 2, *P* < 0.05	72	1	8	29	89.7	95.6	0.911
Jiang¹	2018	circPOLE2 (7)	PE	Microarray	FC > 2, *P* < 0.05	23	12	7	18	76.67	60	0.728
Jiang²	2018	circRNF38 (8)	PE	Microarray	FC > 2, *P* < 0.05	16	9	14	21	53.33	70	0.621
Jin¹	2021	hsa_circNFXL1_009 (9)	PAH	qRT-PCR	FC > 2, *P* < 0.05	19	1	2	20	90.48	95.24	0.941
Jin²	2021	hsa_circMFN2_004 (10)	PAH	qRT-PCR	FC > 2, *P* < 0.05	13	4	8	17	61.9	80.95	0.747
Jin³	2021	hsa_circ_ZNF302 (11)	PAH	qRT-PCR	FC > 2, *P* < 0.05	18	5	3	16	85	76.19	0.847
Jin^4^	2021	hsa_circGSDMD_004 (12)	PAH	qRT-PCR	FC > 2, *P* < 0.05	19	8	2	13	90.04	60	0.731
Jin^5^	2021	hsa_circWDR37_016 (13)	PAH	qRT-PCR	FC > 2, *P* < 0.05	19	16	2	5	90	25	0.822
Li	2021	hsa_circ_0001599 (14)	IS	qRT-PCR	FC > 2, *P* < 0.05	76	12	42	106	64.41	89.83	0.805
Liang¹	2020	circZNF609 (15)	CAD	RT-PCR	*P* < 0.0001	26	9	4	21	86.7	70	0.83
Liang²	2020	circZNF609 (16)	CAD	RT-PCR	*P* < 0.0001	233	66	67	113	77.7	63	0.752
Liang³	2020	circZNF609 (17)	CAD	RT-PCR	*P* < 0.0001	265	80	65	129	80.4	61.5	0.761
Liu	2019	circSep-11 (18)	EH	Microarray	FC > 2, *P* < 0.05	64	29	25	60	72.4	67.3	0.741
Peng	2019	circ HECTD1 (19)	IS	qRT-PCR	*P* < 0.001	116	44	44	116	72.5	72.5	0.814
Sun¹	2020	circDEPDC5 (20)	HF	Microarray	FC > 2, *P* < 0.01	26	18	4	12	86.7	40	0.838
Sun²	2020	circLTBP1 (21)	HF	Microarray	FC > 2, *P* < 0.01	26	16	4	14	86.2	46.7	0.759
Sun³	2020	circMARC2 (22)	HF	Microarray	FC > 2, *P* < 0.01	26	14	4	16	86.7	53.3	0.817
Tian	2019	circMARK3 (23)	AAAD	Microarray	FC > 2, *P* < 0.05	27	4	3	26	90	86.7	0.9344
Tian¹	2021	circSLC8A1 (24)	MI	qRT-PCR	FC > 2, *P* < 0.05	31	6	16	12	66.7	67.1	0.706
Tian²	2021	circNFIX (25)	MI	qRT-PCR	FC > 2, *P* < 0.05	33	4	14	14	71.1	77.8	0.868
Wang¹	2019	circNIPSNAP3A (26)	CAD	Microarray	FC ≥ 1.5, *P* < 0.05	342	133	70	157	83.1	54.3	0.703
Wang²	2019	circSPARC (27)	CAD	Microarray	FC ≥ 1.5, *P* < 0.05	291	112	121	178	70.7	61.4	0.7
Wang³	2019	circNIPSNAP3A+circSPARC (28) 19hsa_circ_0004104	CAD	Microarray	FC ≥ 1.5, *P* < 0.05	317	110	95	180	76.9	62	0.742
Wu¹	2019	circFBXW12 (29)	KD	qRT-PCR	*P* < 0.001	46	22	10	34	82.2	60	0.747
Wu²	2019	circANRIL (30)	KD	qRT-PCR	*P* < 0.05	40	23	16	33	72.3	58.9	0.624
Wu	2020	circMCTP1 (31)	CAD	Microarray	FC > 4, *P* < 0.05	87	21	21	68	81	76.5	0.853
xiong	2021	circNPHP4 (32)	CAD	qRT-PCR	FC ≥ 2, *P* < 0.05	95	21	14	49	87.1	69.7	0.837
Yang	2021	circRNA_104761 (33)	MI	qRT-PCR	FC ≥ 2, *P* < 0.05	26	12	4	48	86.7	80	0.89
Zhang¹	2018	circCACNA1C (34)	PoAF	Microarray	FC ≥ 2, *P* < 0.05	60	65	15	225	79.4	77.6	0.802
Zhang²	2018	circCACNA1C (35)	PoAF	Microarray	FC ≥ 2, *P* < 0.05	50	48	18	168	73.52	77.83	
Zhang¹	2019	circST6GAL1 (36)	IPAH	qRT-PCR	*P* < 0.05	61	1	21	81	74.39	98.78	0.895
Zhang²	2019	circST6GAL1 (37)	IPAH	qRT-PCR	*P* < 0.05	26	4	2	49	94.59	92.45	0.978
Zhang³	2019	circST6GAL1 (38)	IPAH	qRT-PCR	*P* < 0.05	16	3	0	63	1	95.45	0.993
Zhao¹	2017	circROBO2 (39)	CAD	Microarray	FC > 2, *P* < 0.01	119	27	18	88	86.7	76.7	0.872
Zhao²	2017	circSRGAP1 (40)	CAD	Microarray	FC > 2, *P* < 0.01	110	15	27	100	80	86.7	0.82
Zhao³	2017	circROBO2+circSRGAP1 (41) hsa_circ_0098964	CAD	Microarray	FC > 2, *P* < 0.01	113	31	24	84	82.5	73	0.811
Zhao	2020	circLIFR (42)	IS	qRT-PCR	*P* < 0.0001	70	10	5	80	93.3	88.9	0.9505
Zheng	2019	circCHTOP (43)	EH	qRT-PCR	*P* < 0.001	63	29	26	60	70.8	67.4	0.732
Zheng	2020	hsa-circRNA9102-5 (44)	EH	Microarray	FC > 2, *P* < 0.01	64	43	32	53	66.7	55.2	0.62
Zhu	2019	circ-DLGAP4 (45)	IS	qPCR	*P* < 0.001	138	56	32	114	81.2	67.1	0.816
Zuo	2020	circFUNDC1 (46)	AIS	Microarray	FC > 4, *P* < 0.01	18	16	8	26	69.23	61.9	0.6612
Zuo	2020	circFUNDC1+circPDS5B +circCDC14A (47)	AIS	Microarray	FC > 4, *P* < 0.01	215	39	21	97	91	0.715	0.897

Abbreviations: CAD, coronary artery disease; AIS, acute ischemic stroke; IS, ischemic stroke; PE, preeclampsia; HF, heart failure; EH, essential hypertension; IPAH, idiopathic pulmonary arterial hypertension; AAAD, acute stanford type A aortic dissection; KD, kawasaki disease; PoAF, postoperative atrial fibrillation; CVD, cardiovascular disease; circRNA, circular RNA; qPCR, real-time polymerase chain reaction; qRT-PCR, RT-PCR/qPCR combined technique; RT-PCR, reverse transcription-polymerase chain reaction; FC, fold change.

**Table 3:** Assessment of diagnostic accuracy and heterogeneity in subgroup analysis

Subgroups	N	SEN(95%CI)	SPE (95%CI)	LR^+^ (95%CI)	LR^-^(95%CI)	DOR (95%CI)	AUC (95%CI)
ALL	47	0.81 (0.78-0.83),*I²*=78.42,*P*<0.001	0.74 (0.68-0.78),*I²*=86.97,*P*<0.001	3.1 (2.5-3.7)	0.26 (0.22-0.31)	12 (9-16)	0.85 (0.81-0.88)
**CVD**							
CAD	15	0.75 (0.65-0.83),*I²*=77.28,*P*<0.001	0.67 (0.59-0.74),*I²*=83.49,*P*<0.001	2.4 (2.0-2.9)	0.30 (0.25-0.35)	8 (6-11)	0.82 (0.78-0.85)
IS	8	0.83 (0.74-0.89),*I²*=85.63,*P*<0.001	0.81 (0.71-0.88),*I²*=83.73,*P*<0.001	3.9 (2.5-6.2)	0.19 (0.12-0.32)	20 (8-50)	0.89 (0.86-0.91)
**Method**							
qRT-PCR	39	0.81 (0.77-0.84),*I²*=80.35,*P*<0.001	0.74 (0.68-0.80),*I²*=89.89,*P*<0.001	3.0 (2.3-3.9)	0.27 (0.22-0.34)	11 (7-18)	0.84 (0.81-0.87)
qPCR	7	0.82 (0.77-0.86),*I²*=77.89,*P*<0.001	0.68 (0.64-0.72),*I²*=46.97,*P*<0.001	2.6 (2.2-3.0)	0.27 (0.21-0.35)	10 (7-14)	0.78 (0.74-0.81)
**Specimen**							
Blood	27	0.79 (0.75-0.83),*I²*=83.68,*P*<0.001	0.71 (0.65-0.76),*I²*=83.05,*P*<0.001	2.4 (2.0-2.9)	0.31 (0.25-0.39)	8 (5-12)	0.80 (0.76-0.83)
Serum	9	0.85 (0.79-0.90),*I²*=72.66,*P*<0.001	0.88 (0.75-0.94),*I²*=92.34,*P*<0.001	8.0 (3.4-18.8)	0.17 (0.11-0.27)	47 (15-149)	0.91 (0.89-0.94)
Others	11	0.80 (0.77-0.83),*I²*=0.00,*P*<0.001	0.65 (0.56-0.73),*I²*=84.13,*P*<0.001	2.3 (1.8-2.9)	0.31 (0.27-0.36)	7 (5-10)	0.81 (0.78-0.85)
**Sample size**							
<200	30	0.82 (0.78-0.85),*I²*=73.51,*P*<0.001	0.76 (0.68-0.82),*I²*=89.02,*P*<0.001	3.4 (2.3-5.1)	0.25 (0.18-0.34)	14 (7-27)	0.86 (0.82-0.88)
≥200	17	0.79 (0.76-0.83),*I²*=81.82,*P*<0.001	0.70 (0.65-0.75),*I²*=85.81,*P*<0.001	2.5 (2.2-3.0)	0.29 (0.24-0.34)	9 (7-12)	0.82 (0.78-0.85)
**Source of control**							
Healthy	22	0.79 (0.74-0.83),*I²*=85.03,*P*<0.001	0.72 (0.63-0.79),*I²*=86.91,*P*<0.001	2.5 (1.8-3.6)	0.31 (0.23-0.41)	8 (5-15)	0.82 (0.78-0.85)
Non-CVD	25	0.81 (0.78-0.84),*I²*=66.06,*P*<0.001	0.73 (0.68-0.78),*I²*=84.31,*P*<0.001	3.2 (2.5-4.2)	0.24 (0.20-0.31)	13 (8-21)	0.86 (0.82-0.88)
**Year**							
~2019	8	0.79 (0.73-0.84),*I²*=64.24,*P*<0.001	0.77 (0.73-0.81),*I²*=54.87,*P*<0.001	3.5 (2.8-4.3)	0.27 (0.21-0.36)	13 (8-20)	0.84 (0.81-0.87)
2019~	39	0.81 (0.78-0.84),*I²*=82.63,*P*<0.001	0.73 (0.67-0.78),*I²*=88.18,*P*<0.001	2.8 (2.2-3.7)	0.26 (0.21-0.33)	11 (7-17)	0.84 (0.81-0.87)

Abbreviations: CVDs, cardiovascular diseases; CAD, coronary artery disease; IS, ischemic stroke; SEN, sensitivity; SPE, specificity; CI, confidence interval; LR^+^, positive likelihood ratio; LR^-^, negative likelihood ratio; DOR, diagnostic odds ratio; AUC, area under the curve.

**Table 4:** Summary of circRNAs with their potential targets and related pathways

Author	CircRNA	Disease	Control	Expression	Potential target(s)	Related pathways
Bazan2017	circR-284	IS	Non-IS	U	miR-221	↓miR-221
Bu2019	hsa_circ_0001946	CHD	Healthy	U	PARP1	↓cell proliferation, migration and invasion; ↓has-miR-7-5p, ↑PARP1, ↑cell apoptosis; ↓EGFR mRNA, ↓downstream protein kinases
	hsa_circ_0008507	CHD	Healthy	U	/	↑adhesion of peripheral blood leukocytes to blood vessels
	hsa_circ_0000284	CHD	Healthy	D	/	↓endothelial cell proliferation, migration and tube formation, ↓growth, ↓plaque rupture of vascular endothelial cells, ↓adhesion of peripheral blood leukocytes to blood vessels
Chen2020	hsa_circ_0141720	AIS	Healthy	U	/	↑hs-CRP, ↑IL-6, ↑immune cells
Jiang2018	hsa_circ_0004904	PE	Non-PE	U	PAPP-A	↓MREs, ↑PAPP-A, IGF axis
	hsa_circ_0001855	PE	Non-PE	U	PAPP-A	↓MREs, ↑PAPP-A, IGF axis
Liang2020	circZNF609	CAD	Non-CAD	D	AKT1, Smad7	↓cardiac inflammation, ↓atherosclerosis chronic inflammation, ↑anti-inflammation genes, ↓pro-inflammatory cytokines, ↑anti-inflammatory cytokines; ↓miR-138-5p, ↑AKT1, ↓IL-6; ↓miR-150-5p, ↓miR-615-5p
Liu2019	hsa_circ_01269919	EH	Healthy	U	miR-10a-5p	↑inflammation, ↓miR-10a-5
Peng2019	circRNA HECTD1	AIS	Non-AIS	U	TRAF3	↓autophagy; ↑pro-inflammatory cytokines, ↑inflammation, ↑immune responses; ↓TRAF3, ↓miR-142, ↓astrocyte activation; ↓miR-133b, ↑infarct
Sun2020	hsa_circ_0062960	HF	Non-HF	U	/	platelet activity
	hsa_circ_0053919	HF	Non-HF	U	/	platelet activity
	hsa_circ_0112085	HF	Non-HF	U	/	platelet activity
Tian2019	circMARK	AAAD	Non-AAAD	U	Fgr	↓apoptosis, ↑inflammation, ↓p53, ↑Fgr, ↑HASMC
Wang2019	hsa_circ_0001879	CAD	Healthy	U	/	PI3K/Akt pathway
	hsa_circ_0004104	CAD	Healthy	U	MAPK, TGF-β	PI3K/Akt pathway, ↑cardiac fibrosis; ↑IDO1, ↑MMP8, ↑CD40, ↓ApoA I, ↓RNASE1
Wu2019	hsa_circ_0123996	KD	Healthy	U	/	/
	circANRIL	KD	Healthy	D	PES1	↑antiatherogenic cell functions; ↑apoptosis, ↓proliferation, ↓excessive the proliferation of VSMC; ↓protein translation rate, ↓cell growth
Wu2020	hsa_circ_0005540	CAD	Non-CAD	U	miR-221, miR-145	regulate endothelial cells, migration, capillary tube formation
Zhang2018	hsa_circRNA_025016	PoAF	No-PoAF	U	/	regulate melanogenesis, insulin and thyroid hormone secretion
Zhang2019	circ_0068481	IPAH	Healthy	U	/	/
Zhao2017	hsa_circ_0124644	CAD	Non-CAD	U	/	cell apoptosis
	hsa_circ_0098964	CAD	Non-CAD	U	/	/
Zhao2020	circ_0072309	AIS	Non-AIS	D	miR-100, hsa-miR-519e-5p, hsa-miR-516b-5p, miR-492	↑apoptosis, ↓cell proliferation, migration; ↑necrosis, ↑apoptosis of SMC, PI3K/Akt pathway; ↑miR-100, ↑mTOR signal pathway; ↑hsa-miR-519e-5p, ↑hsa-miR-516b-5p,↑miR-492
Zheng2019	hsa_circ_0014243	EH	Healthy	U	hsa-miR-10a-5p	↓hsa-miR-10a-5p
Zheng2019	hsa-circRNA9102-5	EH	Healthy	U	hsa-miR-150-5p	↑endothelial dysfunction, ↓angiogenesis, ↓hsa-miR-150-5p
Zhu2019	circ-DLGAP4	AIS	Non-AIS	D	miR-143	↓cardiomyocytes apoptosis, ↓inflammation; ↓miR-143
Zuo2020	circFUNDC1	AIS	Non-AIS	U	FUNDC1	mitophagy
Zuo2020		AIS	Non-AIS	U	/	/

Abbreviations: PARP1, poly (ADP-ribose) polymerase 1; EGFR: epidermal growth factor receptor; hs-CRP, high-sensitivity C relative protein; IL-6, interleukin 6; MREs, miRNA recognition elements; PAPP-A, pregnancy-associated plasma protein A; AKT1, a member of the three serine/threonine protein kinase family; Smad7, a negative regulator of TGF-β signaling; TRAF3, the tumor necrosis factor receptor-associated factor 3; Fgr, tyrosine-protein kinase; HASMC: human aortic smooth muscle cells; MAPK: mitogen-activated protein kinase; IDO1/MMP8/CD40, atherosclerosis-susceptible genes; PES1: pescadillo homologue 1; VSMC: vascular smooth muscle cell; SMC: smooth muscle cells; ApoA I/RNASE1, anti-atherosclerosis genes; D, downregulated; U, upregulated.
