# Supplementary material for: Diagnostic value of circRNAs as effective biomarkers in human cardiovascular disease: an updated meta-analysis
Source: Int J Med Sci. 2022 Feb 7;19(3):446–59. doi: 10.7150/ijms.67094 (PMC8964318; doi:10.7150/ijms.67094)

Supplementary figure S1. Summary receiver operator characteristic (SROC) curves based on circRNAs in Subgroup analyses. A. CAD; B. IS; C. Blood; D. Serum; E. qRT-PCR; F. qPCR; G. Sample size<200; H. Sample size≥200; I. Healthy; J. non-CVD ; K. Before 2019; L. After 2019.

Supplementary Figure S1A

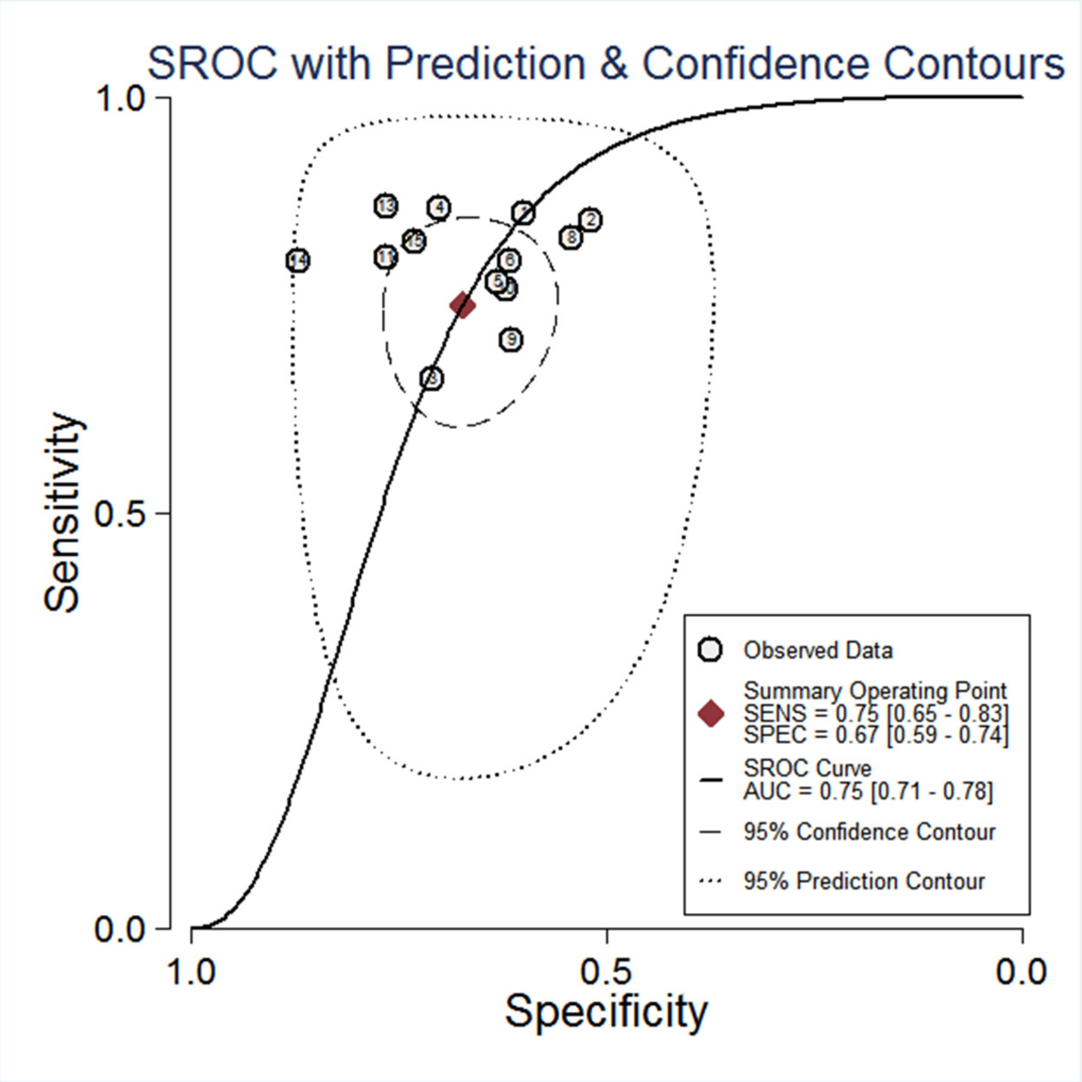

Supplementary Figure S1B

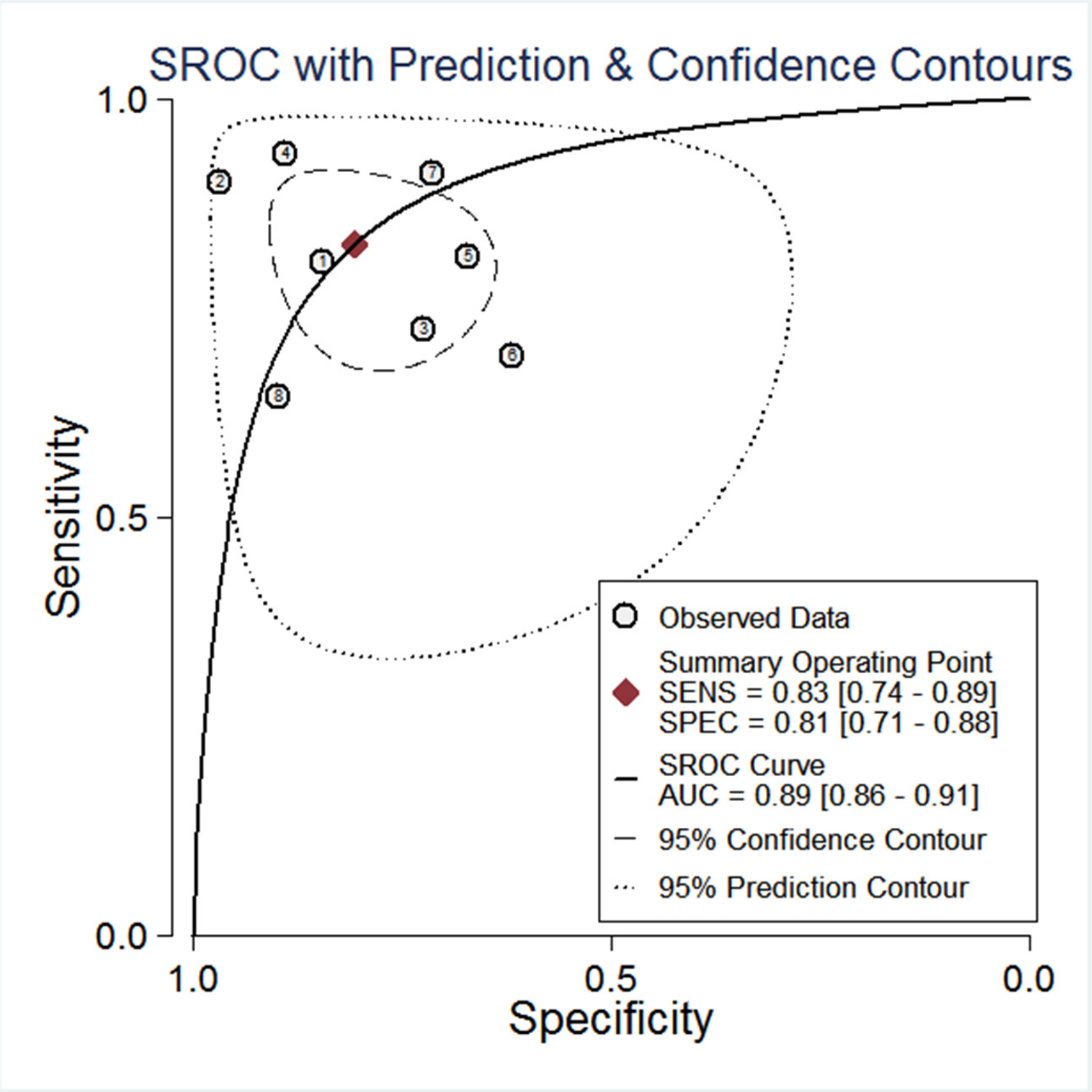

Supplementary Figure S1C

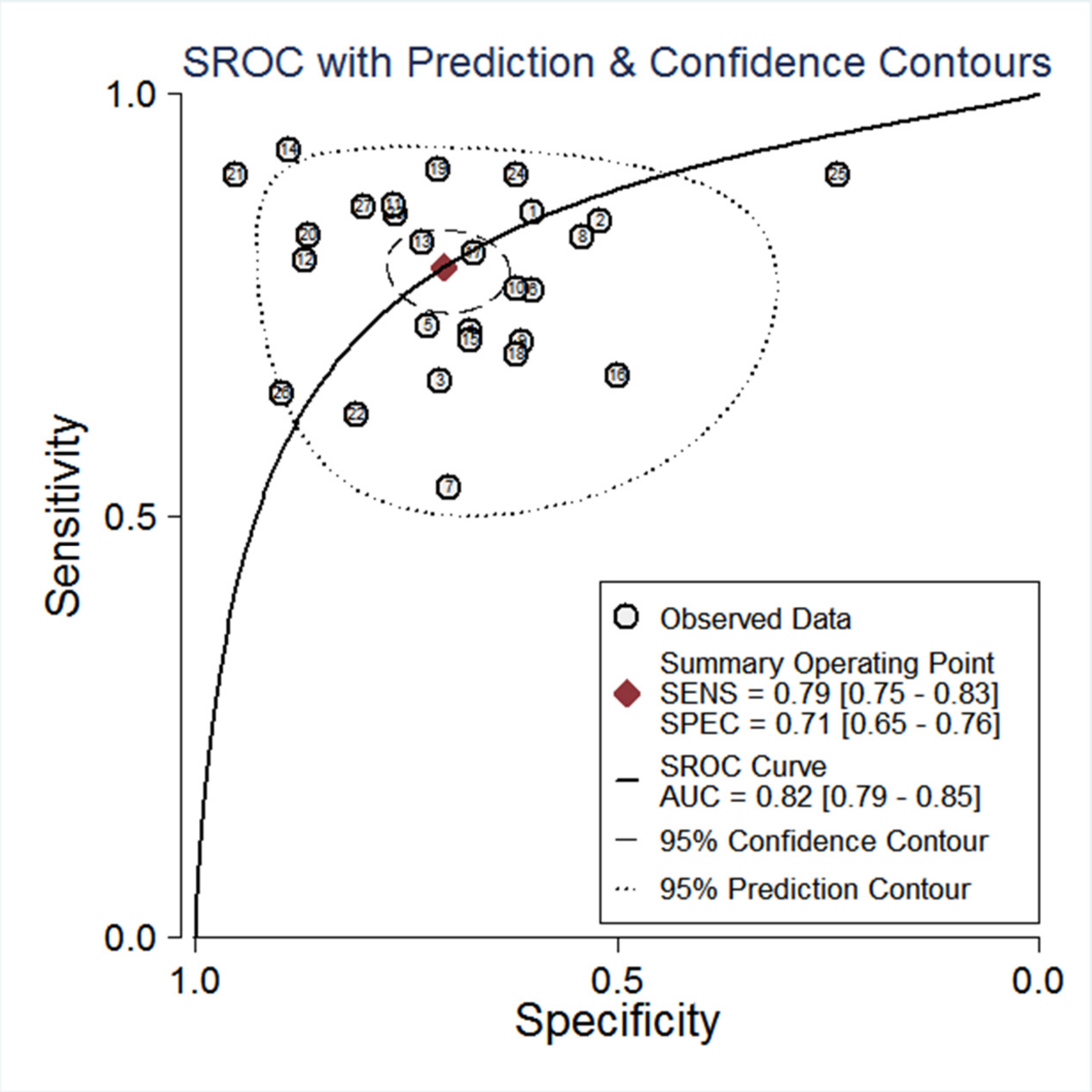

Supplementary Figure S1D

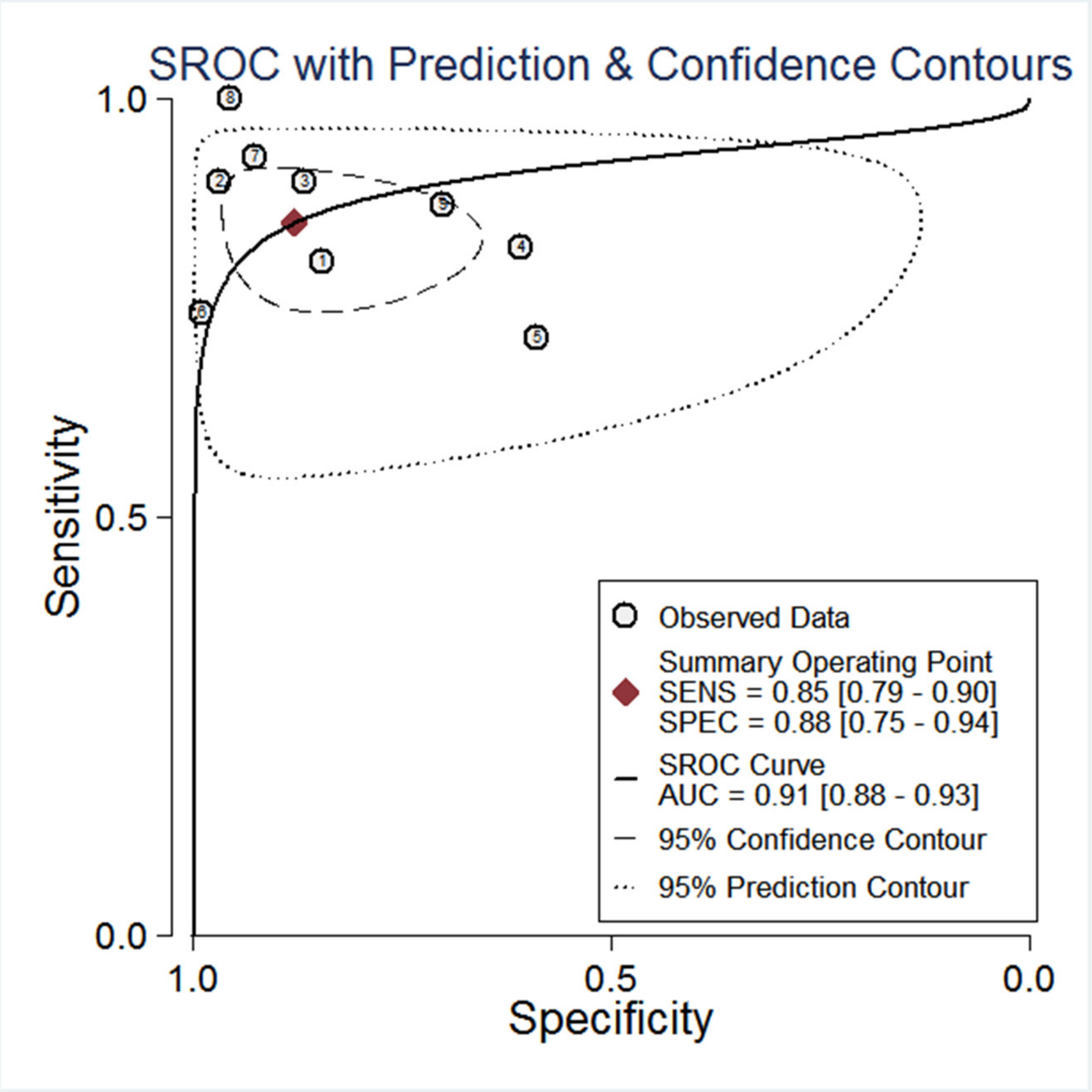

Supplementary Figure S1E

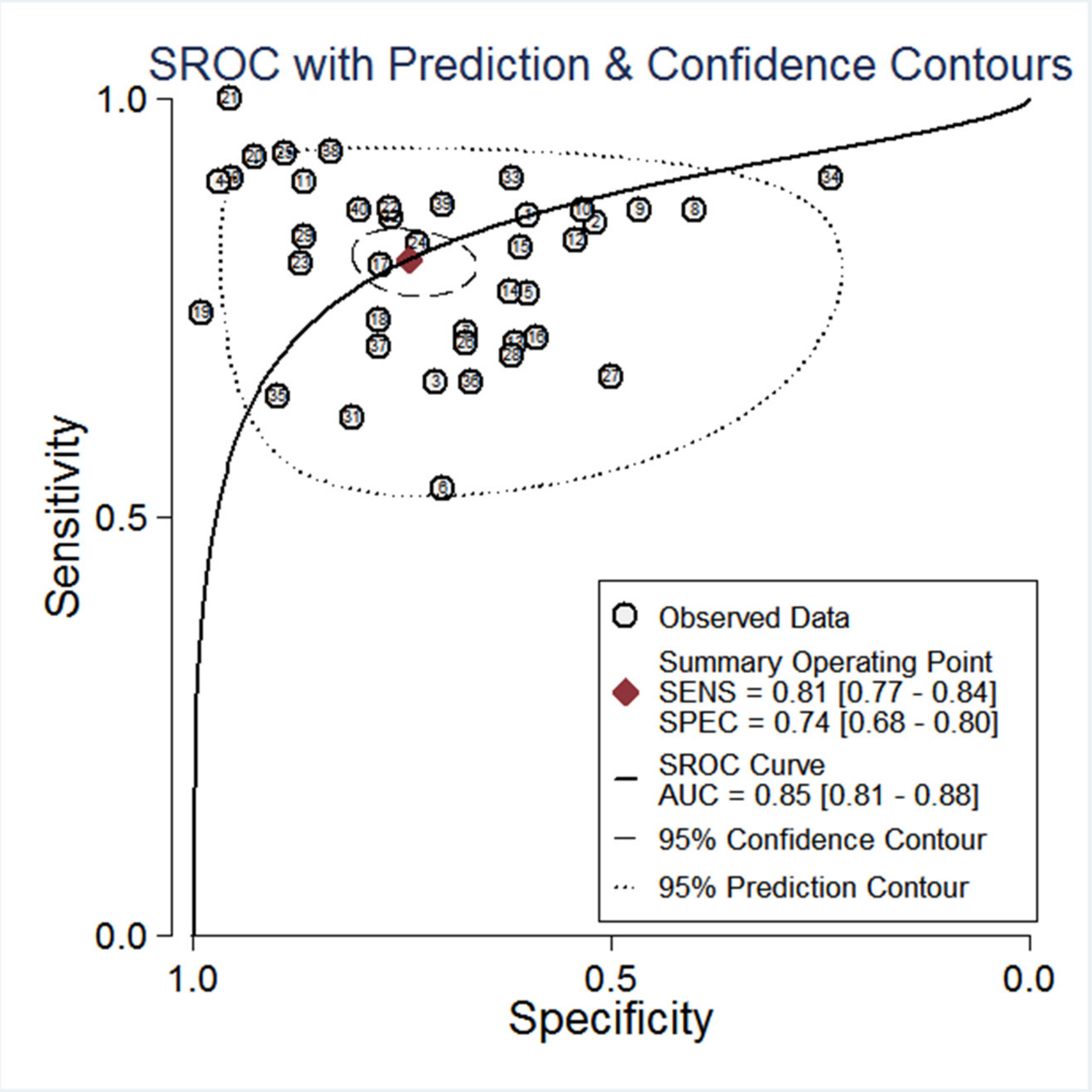

Supplementary Figure S1F

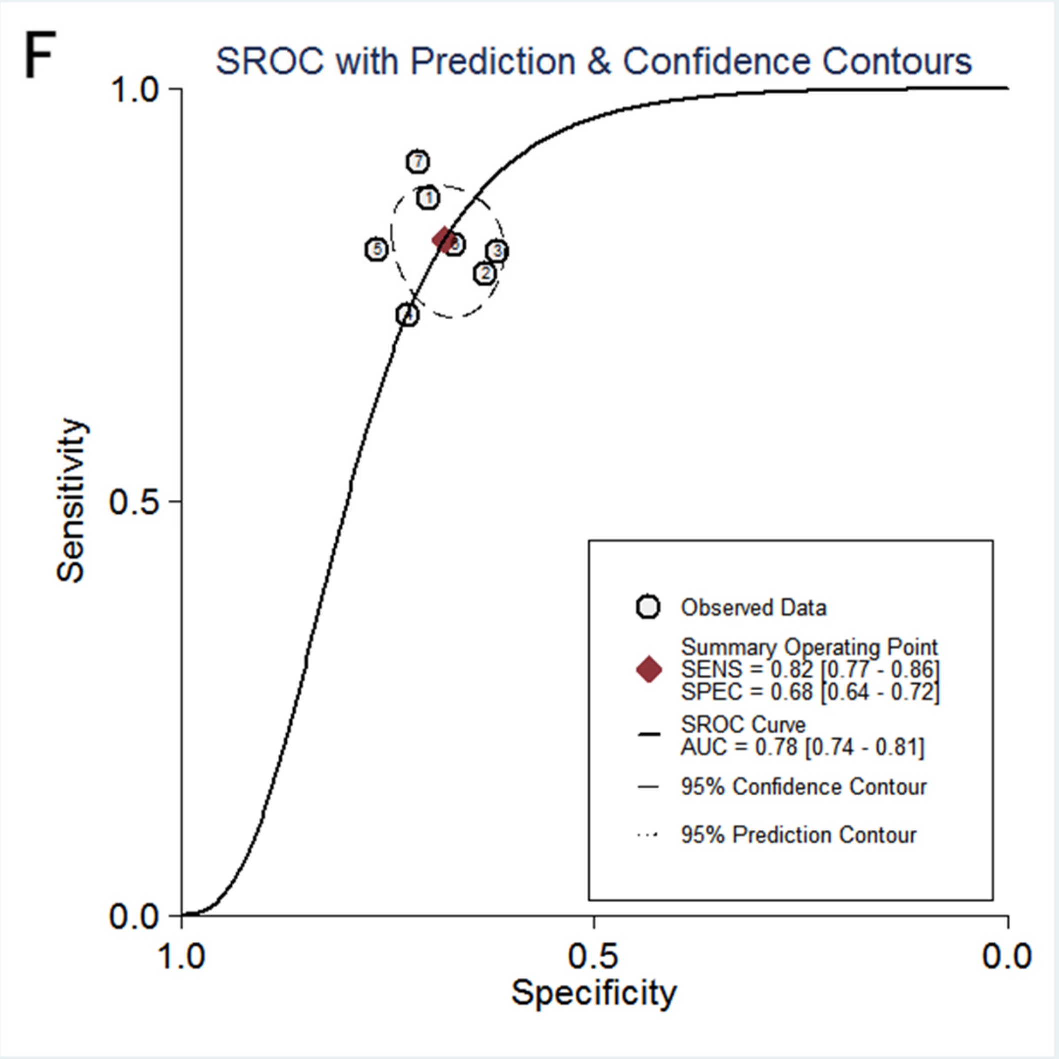

Supplementary Figure S1G

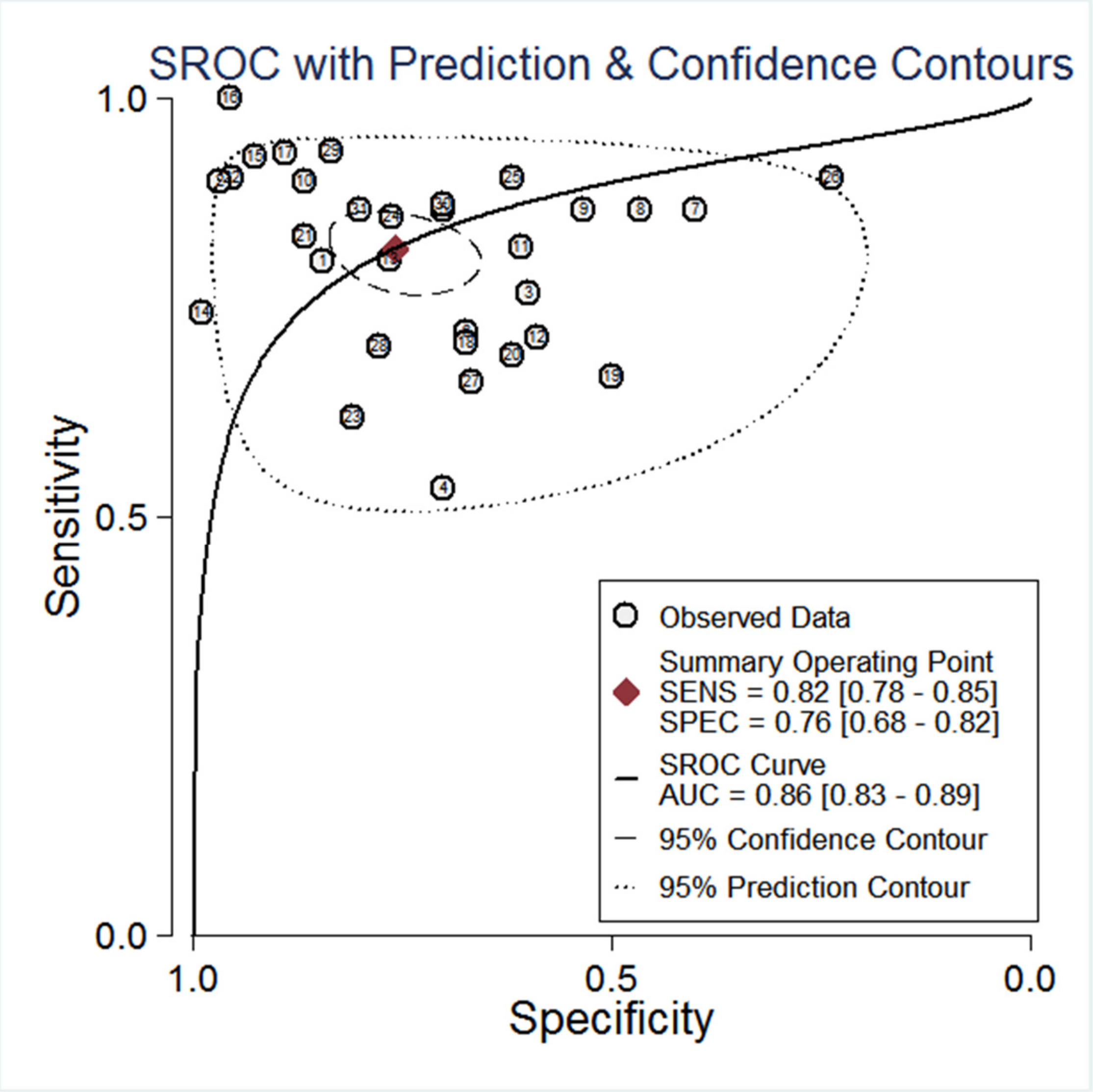

Supplementary Figure S1H

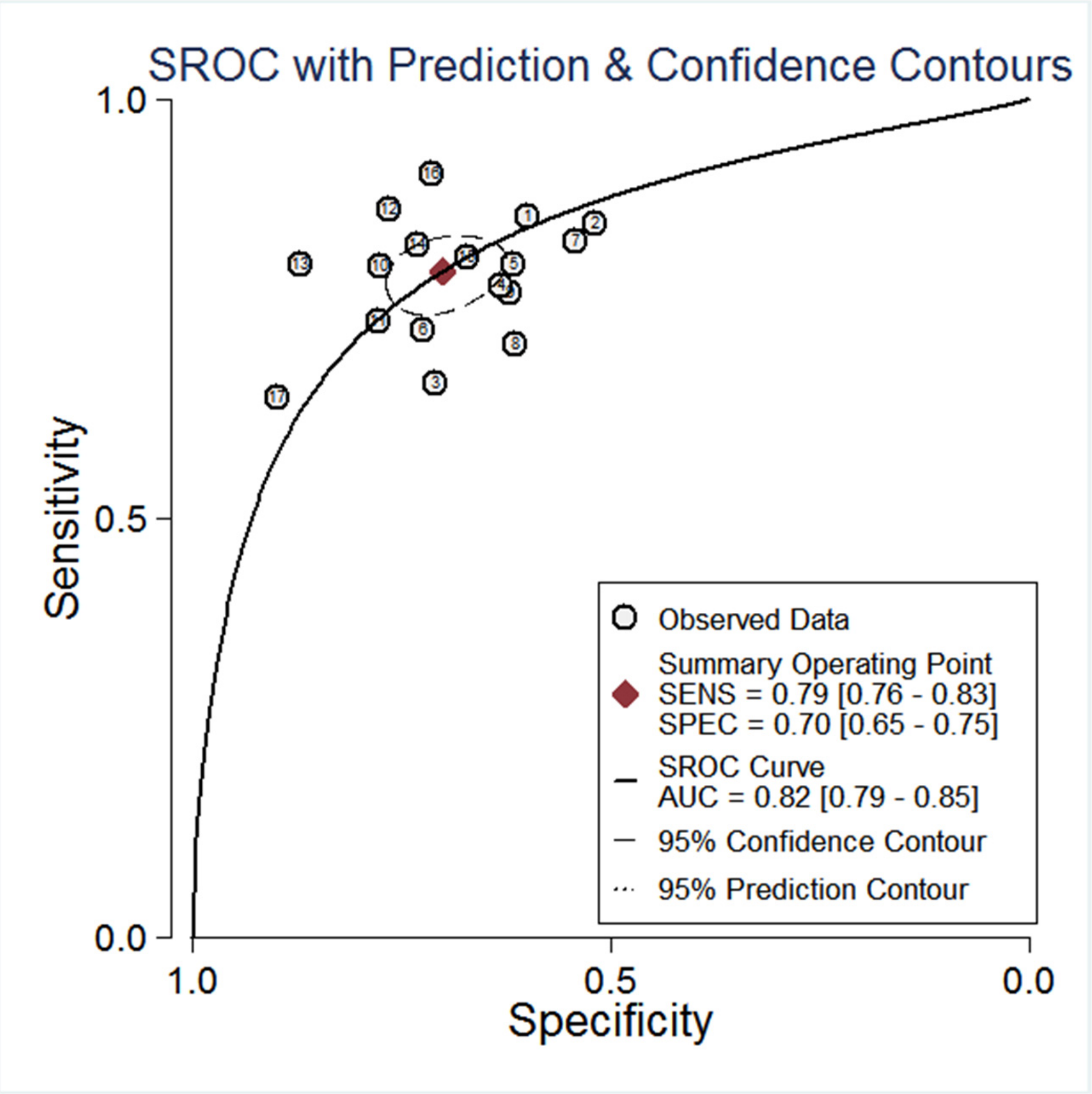

Supplementary Figure S1I

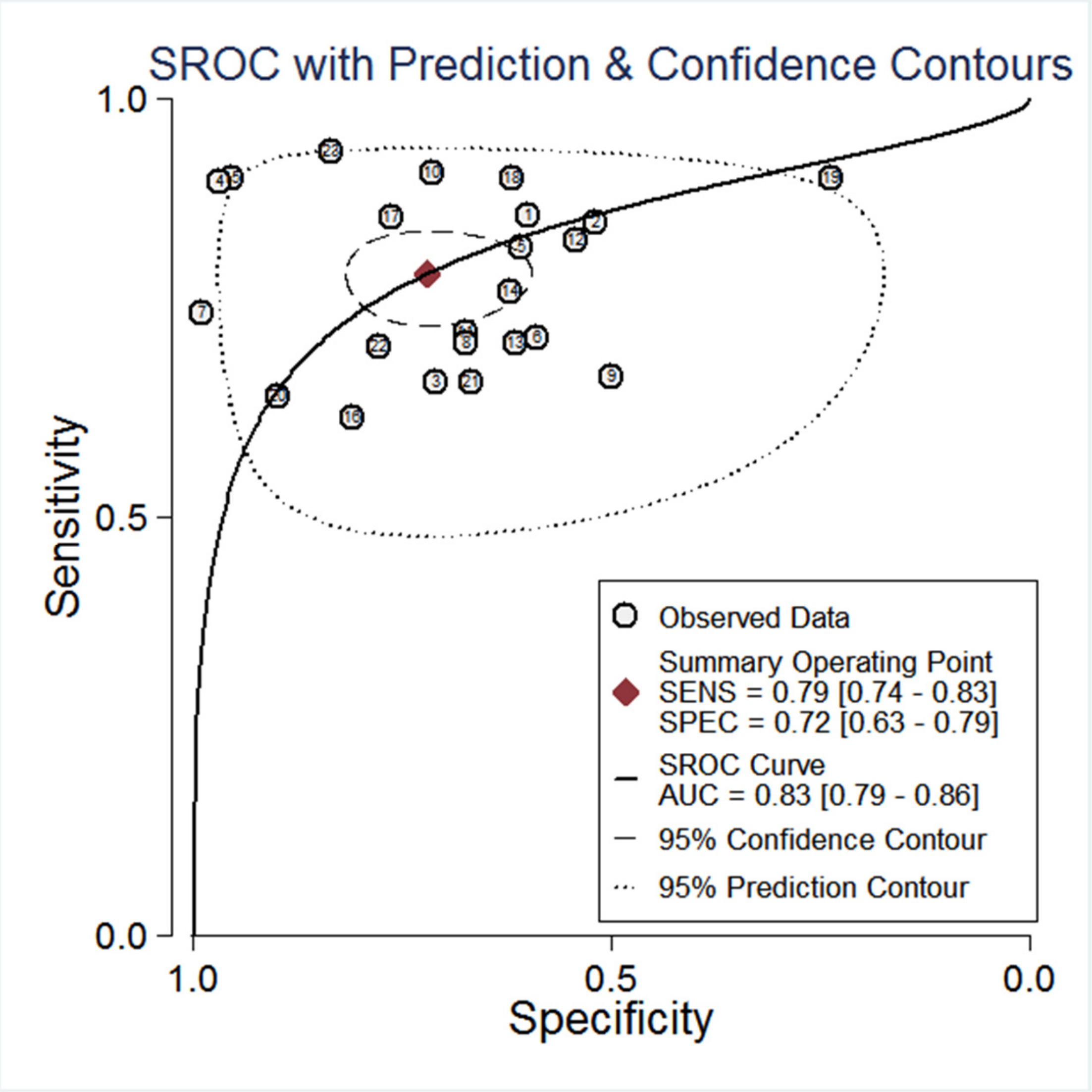

Supplementary Figure S1J

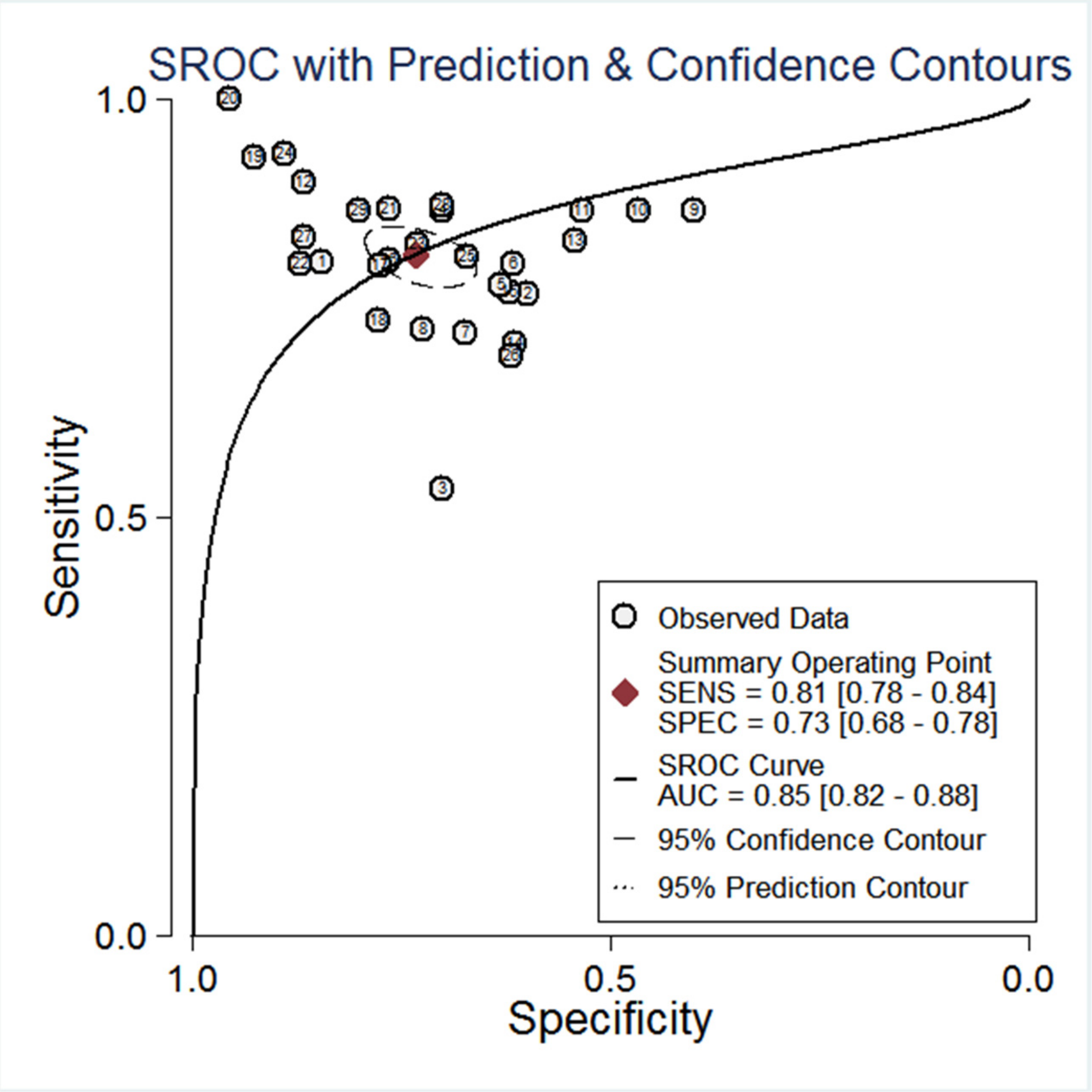

Supplementary Figure S1K

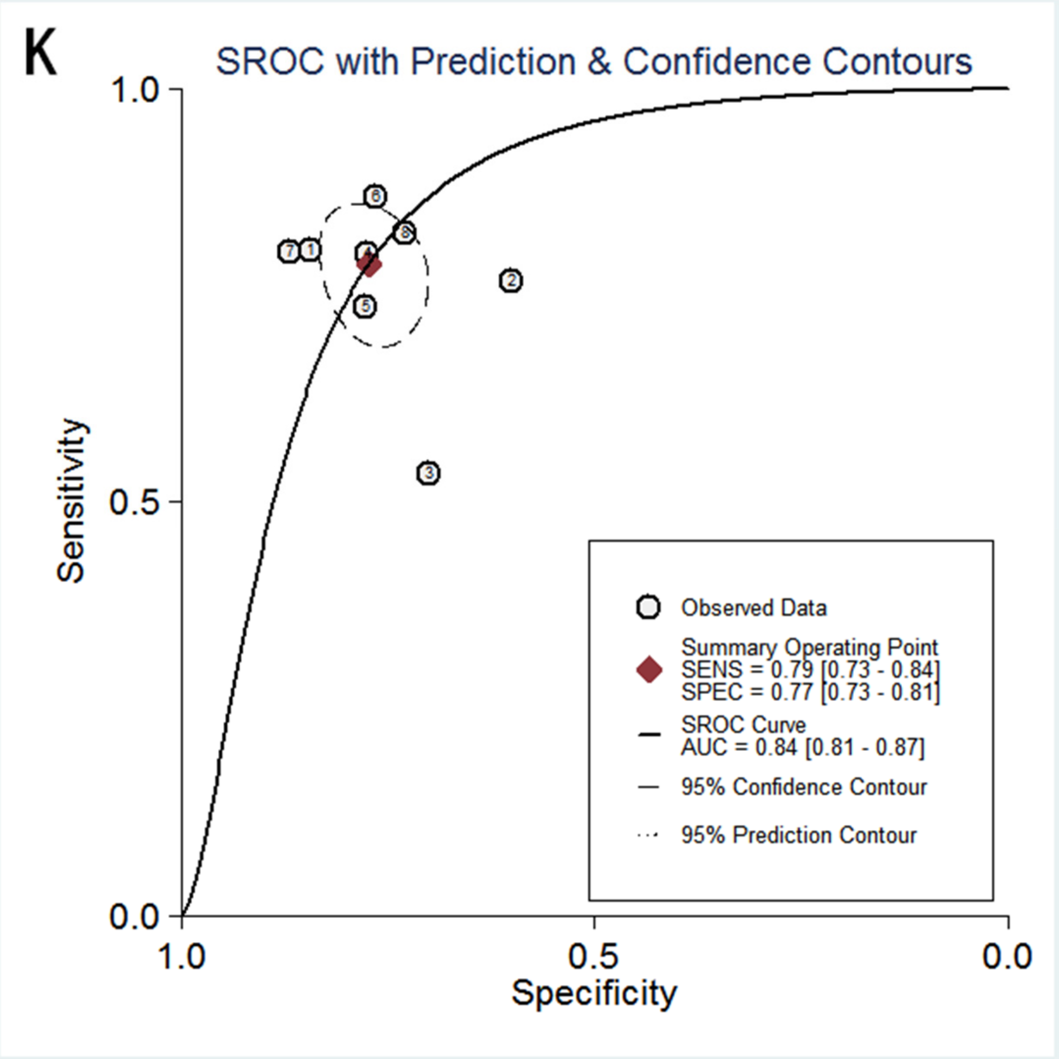

Supplementary Figure S1L

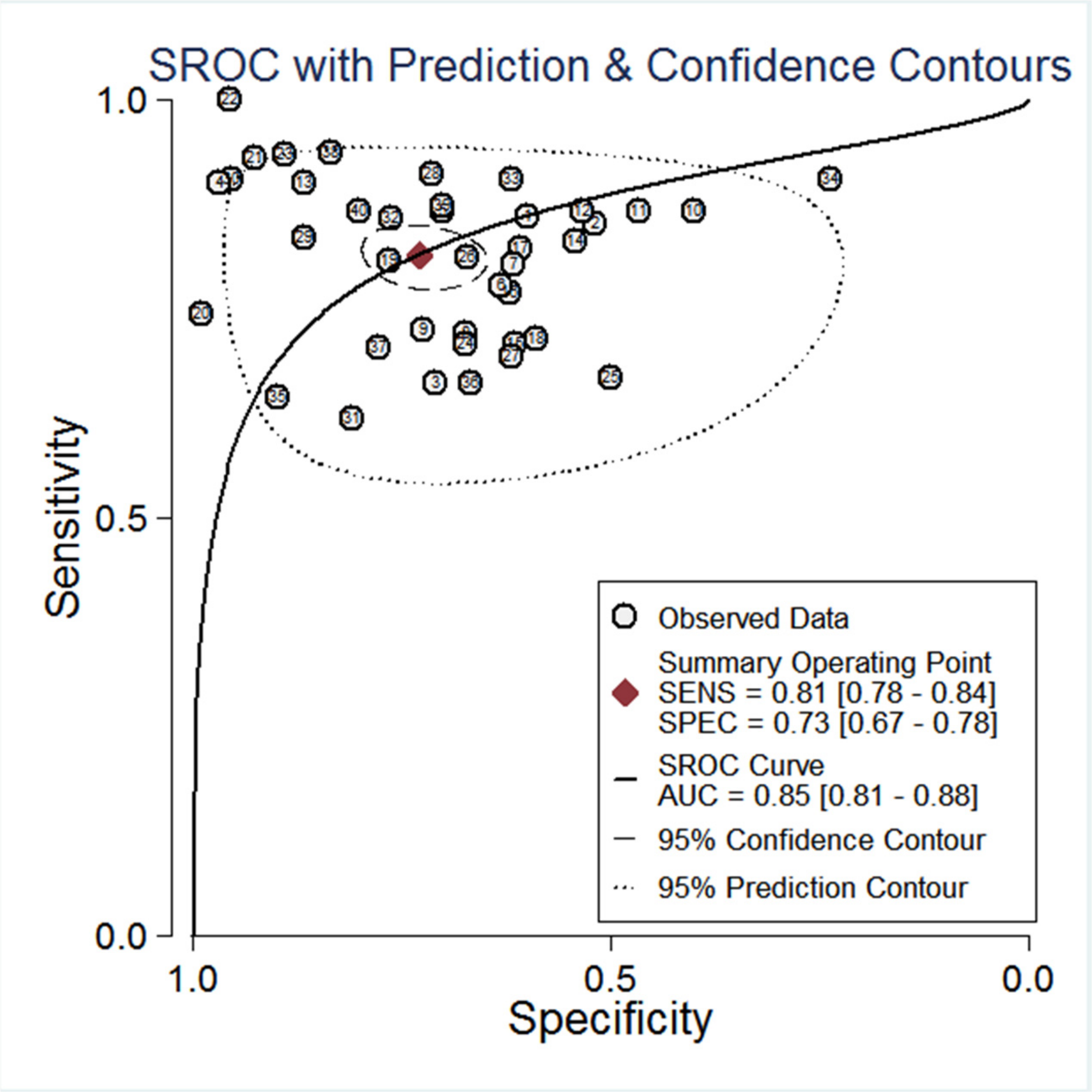

Supplement: Supplementary file 1 — Supplementary figure. [file ijmsv19p0446s1.pdf]
